# Follicle-like niches outside the cortex? 3D phase-contrast µCT revealed medullary B cell nodules in mucosa-draining lymph nodes

**DOI:** 10.3389/fimmu.2025.1674997

**Published:** 2025-11-19

**Authors:** Paul Schütz, Florian L. Schwarzenberg, Lennert J. Weber, Jörg U. Hammel, Bente Siebels, Paula Nissen, Nenya Leising, Katja J. Jarick, Bernd Walkenfort, Sarah C. Irvine, Jasmin Bartl, Julia Herzen, Christian Lohr, Clemens Wülfing, Stephan Henne

**Affiliations:** 1 INI-Research, Group for Interdisciplinary Neurobiology and Immunology, University of Hamburg, Hamburg, Germany; 2 Institute for Materials Physics, Helmholtz-Zentrum Hereon, Geesthacht, Germany; 3 Section Mass Spectrometry and Proteomics, Center for Diagnostics, University Medical-Center Hamburg-Eppendorf, Hamburg, Germany; 4 Imaging Center Essen (IMCES), Electron Microscopy Unit (EMU), Medical Faculty, University of Duisburg-Essen, Essen, Germany; 5 Biomedical Imaging Physics, TUM School of Natural Sciences and Munich Institute of Biomedical Engineering, Technical University of Munich, Garching, Germany; 6 Division of Neurophysiology, University of Hamburg, Hamburg, Germany

**Keywords:** lymph node niches, mucosal immunity, high endothelial venule (HEV), X-ray microtomography, aging, B cell microenvironment, lymphocyte homing, medulla

## Abstract

This study identifies and characterizes previously unrecognized medullary B cell niches within murine mucosa-draining lymph nodes (LNs), challenging the conventional understanding of LN architecture. Utilizing advanced imaging techniques, including synchrotron radiation-based phase-contrast micro-computed tomography (SRµCT), correlated high-resolution electron microscopy and immunohistochemistry (IHC), we revealed spherical to ovoid structures termed *nodules*, being distinct lymphoid compartments consistently localized in the medullary region of mandibular and other mucosa-draining LNs. These nodules were primarily composed of unswitched, non-proliferative CD45R^+^ B cells expressing IgD and IgM, lacking germinal center features or typical activation markers. They were seamlessly integrated into the medullary architecture, surrounded by LYVE-1^+^ lymphatic endothelial cells, and situated in close proximity to medullary high endothelial venules (HEVs), revealed by PNAd staining. Under steady-state conditions, this has not been previously observed in the medullary compartment of LNs but is likely facilitating nodule-like B cell aggregation in mucosa-draining LNs due to sustained low level antigenic stimulation common in mucosal environments and is underpinned by proteomics. Additionally, the nodules displayed a capillary network that closely resembles the vascularization seen in conventional B cell follicles revealed by SRµCT. Nodule formation occured between two and four weeks postnatally, thus emerging later than B cell follicles, and their abundance showed a tendency to increase with age. Functionally, these nodules appear to represent a quiescent B cell niche, potentially supporting B cell homeostasis, tolerance, or memory-like readiness, and are distinct from pathological hyperplasias. Their preservation in aged LNs, coupled with the absence of lipomatosis, suggests a role in maintaining structural integrity and immune readiness through persistent B cell-stromal interactions. This research challenges the established paradigm of LN microarchitecture and suggests specialized niches for B cell function and lymphocyte trafficking in regions subject to constant antigenic exposure.

## Introduction

Lymph nodes (LNs) are secondary lymphoid organs (SLOs) strategically positioned throughout the body. During embryogenesis, LN anlagen form at the interface of blood and lymphatic vessels, and are rapidly colonized by mature lymphocytes after birth. T cells (CCR7^+^) and B cells (CXCR5^+^) enter via newly formed high endothelial venules (HEVs) and afferent lymphatics with T cells being present from postnatal day one whereas B cells become detectable around day seven, with follicles forming by week two (1). Distinct T and B cell zones emerge due to the differentiation of stromal subsets. Fibroblastic reticular cells (FRCs) form the T-zone reticulum and guide T cell trafficking with CCL19/CCL21, while B cell follicles arise around perivascular stromal niches. Follicular dendritic cells (FDCs) develop in B cell areas under postnatal cues, which drive the stromal precursors to adopt a CXCL13^+^ FDC fate. Further, both LTα_1_β_2_ and TNF signals from B cells and dendritic cells (DCs) are required to organize the newly formed FDC network toward its mature form (2). Within B cell follicles a defined subset of B cells is localized. Follicular (FO) B cells reside in follicles and express high IgD and CD23 with moderate IgM (3–6). Germinal center (GC) B cells arise during T-dependent responses within follicles. They are typically identified by GL7^+^, CD95^hi^, IgD^lo^, and CD38^lo^. These cells are rapidly proliferating and undergoing somatic hypermutation, giving rise to class-switched memory and plasma cells (7–11). Plasma cells (PCs) are terminally differentiated antibody-secreting cells with the hallmark surface marker being CD138. Mature PCs lose ordinary B cell markers thus are commonly characterized as CD138^hi^ and CD45R^lo^ (12, 13). Memory B cells represent a heterogeneous population of B cells that protect the host upon antigen (Ag) re-encounter. Memory B cells lack a single universal marker, but can be defined as antigen-experienced, often class-switched (IgG/A) cells that can be IgD^-^ or IgM^+^. In practice, memory B cells are often defined functionally (long-lived, rapid responders) with the supporting expression of CD80, CD73, PD-L2. No single marker uniformly captures all memory B cells, so multi-parameter phenotyping must be used (14–17).

In line with B cell follicle maturation, HEVs transdifferentiate between week one and two postnatally. HEVs are specialized postcapillary venules distinguished by their unique morphology. The endothelial cells lining these vessels are characteristically tall and cuboidal, which contrasts with the flattened, squamous endothelium of typical venules (18). HEVs are predominantly located in the T cell zones of the paracortex and the interfollicular areas adjacent to B cell follicles in the cortex (19). However, their structure gradually transitions as they approach the medulla of the LN, where they resemble regular venules. In these regions, endothelial cells near the medullary sinuses adopt a flattened shape, while those facing the T cell zones retain their cuboidal form (20). Within the medulla, HEVs have transitioned completely into ordinary venules. Essential for lymphocyte homing, HEVs facilitate lymphocyte passage through the endothelium (19, 21, 22). In order to avoid fluid leakage during cell migration, the endothelium adapted into a cuboidal shape (23). Lymphocyte trafficking across HEVs is regulated by specific adhesion molecules that slow lymphocytes at targeted sites before they enter the LN (24). Among these molecules, peripheral node addressin (PNAd) marks the sulfated O-glycan epitopes of mucin-like glycoproteins such as CD34 and GlyCAM-1, which constitute the functional ligand state to bind L-selectin-bearing lymphocytes (25). This ligand-competent form is recognized by MECA-79 and is widely used for HEV identification in LNs (21, 26). The initiated slowing mechanism is critical for the recruitment of naïve B and T cells, memory T cells, and precursor conventional dendritic cells (pre-cDCs), enabling their exit from the bloodstream and entry into the LN parenchyma. Facilitating the rolling adhesion and transmigration is most likely supported by capillaries. The friction between lymphocytes and the capillary vessel wall slows the lymphocytes, allowing erythrocytes to build up and surge through at the transition into a wide-diameter HEV, thus pushing lymphocytes toward the vessel walls (27, 28). This process has been hypothesized to occur, for instance, directly at the B cell follicle borders, where the dense capillary networks transition into HEVs (29). Endothelial LT-βR signaling is critical for the formation of HEVs shown by studies with mice lacking LT-βR on blood endothelium. LT-βR^-^ vessels showed flattened HEVs with low adhesion molecule and chemokine expression which severely impaired lymphocyte homing (30).

The outermost layer, the cortex – comprising B cell follicles and interfollicular regions – encases the medulla and the deep cortical units (DCUs) of the paracortex, where T cells reside (31). Each LN drains a specific area, allowing for constant surveillance of the local tissue homeostasis. Changes in the cellular and molecular composition of afferent lymph fluid carries information indicative of potential pathogenic dangers or altered cell behavior. These changes indicate pathogenic invasion, viral infection, or tumor development due to altered cell behavior. Upon detection of changes in lymph composition, adequate immunological responses unfold by induction of a cell-mediated T cell response or an antigen-mediated B cell activation. Critical immune interactions occur at the T-B cell interface within the interfollicular zones, where B cells engage with T helper cells. This interaction is essential for B cell activation, leading to proliferation, somatic hypermutation, and immunoglobulin production and eventually induced immunity (32). Strong immune responses but also age and chronic processes can alter the LN microarchitecture substantially throughout life. Under certain conditions, lipopolysaccharide (LPS) and Toll-like receptor 4 (TLR4) signaling lead to the downregulation of CXCL13, which drastically alters lymphocyte migratory behavior and disrupts the organization of B cell follicles (33). Likewise, aging is associated with a gradual disorganization of LN compartments. Studies of aged mice show that B cell follicles lose their well-defined boundaries and B cells spread into broader areas of the LN (34). Generally, LN remodeling is a complex process that is highly dependent on the nature of the infection and the cellular and molecular activities taking place within the LN.

B cells are one of the key players in immune surveillance, defending the body against invading pathogens. Mature naïve B cells, identified by expression of IgM and IgD, differentiate upon antigen contact into either memory B cells or antibody-secreting plasma cells (35). However, beyond the B cell subtypes associated with follicles, several additional B cell subsets exist, two of which are particularly relevant to this study: First, “Regulatory” B cells (Bregs) are defined by function rather than a single lineage, with all being potent mediators of immunosuppression through inhibitory mechanisms predominantly involving IL-10 (36). Multiple B cell subsets can act as Bregs. Notable mouse Breg phenotypes include B10-cells, transitional 2-marginal zone precursor B cells (T2MZB), and certain plasma cells/plasmablasts. Most IL-10^+^ Bregs share CD1d^hi^ expression, while co-expression of CD1d^hi^ and CD5^+^ characterizes the classic B10 subset. However, it is important to note that *any* B cell can become regulatory when induced, so phenotypic definitions are not exclusive (37–39). Second, age‐associated B cells (ABCs) are a distinct B cell subset first described in aged female mice. Phenotypically, ABCs overlap only partially with other subsets regarding function and surface marker profiles. Therefore, ABCs resemble a functionally distinct specialized “memory‐like” subset. Their immunoglobulin genes often carry somatic mutations and diverse specificities, but they arise in the context of chronic stimulation or age rather than classical germinal‐center memory pathways (40–42). Importantly, any named B cell subtype markers in this text cover only a part of the whole list. Often multiple markers in co-expression are needed to fully recognize a specific subset of B cells (**Table 1**).

**Table 1:** Representative B cell subtypes and their associated surface marker profiles.

B cell subset	Phentotypic markers (mouse)	References
B-1 cells	CD45R^lo^, CD19^+^, CD11b^+^, IgM^hi^, IgD^lo^	(49–52)
Follicular (FO) B cells	CD45R^+^, CD19^+^, IgM^lo^ IgD^hi^, CD23^hi^, CD21^int^	(3–6)
Transitional T1 & T2 B cells	T1 & T2: CD45R^+^, CD19^+^, IgM^hi^, CD93^+^ T1: IgD^lo^, CD23^-^, CD21^-^ T2: IgD^hi^, CD23^+^, CD21^+^	(53–55)
Transitional T3 B cells	CD45R^+^, CD19^+^, IgM^lo^, IgD^hi^, CD23^+^, CD21^+^, CD93^+^ (anergic, post-T2 stage)	(53–55)
Germinal Center (GC) B cells	CD45R^+^, CD19^+^, GL7^+^, CD95^hi^, IgD^-^, CD38^lo^, PNA^+^	(7, 8)
Plasma cells	CD138^hi^, CD19^lo/-^, CD45R^lo/-^ (often Ly6C^+^, Sca-1^+^, TACI^+^)	(12, 13)
Memory B cells (class-switched)	CD19^+^, CD80^+^, PD-L2^+^, CD73^+^, IgG^+^ or IgA^+^	(14–17)
Memory B cells (unswitched)	CD19^+^, IgM^+^; either CD80^+^, PD-L2^+^, or CD80^-^, PD-L2^-^, IgD^+^	(46, 56, 57)
Regulatory B cells (Bregs)	B10 cells: CD1d^hi^, CD5^+^, IL-10^+^ T2MZP: CD19^+^, CD21^hi^, CD23^hi^, CD24^hi^, IgM^hi^, IgD^hi^, IL-10^+^	(37–39)
Age associated B cells (ABCs)	CD19^+^, CD11c^hi^, CD21^-^, CD23^-^, IgM^+^, IgD^+^	(40–42)

This is not an exhaustive list of subtype-specific markers. Accurate B cell phenotyping often requires the combined interpretation of multiple markers in context. T2MZP, Transitional 2-marginal zone precursor B cell

This study focuses on a subset of LNs, which are situated near epithelial border tissues like mucosa. These LNs, often referred to as mucosa-draining LNs, are unique in the way that they are prone to constant pathogen contact. Ongoing pathogenic burden is often associated with chronic inflammatory responses and diseases. However, regulatory processes can counteract chronic inflammation especially in physiological conditions like constant interactions with the commensal microbiota within the intestinal tract (43–45). In this context a detailed understanding of lymphocyte function and the specialized niches they occupy within LNs is particularly important. Yet, site-specific diversity often remains underappreciated, particularly reflected by the generalized treatment of skin-draining and mucosa-draining LNs in immunological studies, neglecting their distinct antigenic environments. While molecular and cellular differences between these LN subsets are increasingly acknowledged and extensively studied, particularly regarding specialized immunological responses like mucosal immunity, lymphocyte homing, and memory effector responses (46–48), relatively little effort has been directed toward investigating whether these functional distinctions are also reflected morphologically.

Here, we aimed to investigate precisely this morphological manifestation of functional diversity. Additionally, given that the continuous antigen exposure likely influences immune architecture over time, we also sought to explore potential age-associated structural changes and whether these differ between skin-draining and mucosa-draining LNs. Utilizing whole-organ, high-resolution synchrotron radiation-based micro-computed tomography (SRµCT) imaging at an isotropic resolution of 1.8 µm per pixel, we revealed previously undescribed medullary niches within mucosa-draining LNs. These spherical structures, termed *nodules*, harbor quiescent B cells and are associated with medullary HEVs under steady-state conditions, directly challenging canonical and generalized models of LN architecture.

For clarity and consistency, we propose the following terminology for LNs and their respective drainage areas. The LN nomenclature used in this study follows the classification established by Van den Broeck et al. (58). Furthermore, LNs located near the skin will be referred to as *skin-draining LNs* (also known as superficial or peripheral LNs), while LNs associated with internal organs will be termed *mucosa-draining LNs*. In mice, all deep LNs are anatomically situated adjacent to either the respiratory, gastrointestinal, or genitourinary tracts, each of which is lined by a mucosal epithelium. It should be noted, however, that some LNs may occupy transitional anatomical regions, making a strict categorization into one of these two groups occasionally ambiguous. Mandibular LNs exemplify this ambiguity due to their anatomical position and diverse drainage functions.

## Material and methods

### Animals

Animals used for this study were C57BL/6J mice. Housing and dissection of organs was carried out at the Division of Neurophysiology of the biological department, University of Hamburg and in accordance with European Union’s and local welfare guidelines (Behörde für Gesundheit und Verbraucherschutz, Hamburg, Germany; GZ G21305/591-00.33). Prior to organ dissection, mice were deeply anesthetized using isoflurane (5% mixed with 1 L/min O_2_) followed by a heart puncture.

### Immunohistochemistry

For systematic screening of nodules within lymph nodes (LNs) of different body positions we examined four female mice (15–20 weeks) in total resulting in four LNs for single LN clusters e.g. caudal mesenteric LN and eight LNs for paired LN clusters e.g. mandibular LN. For a detailed summary of mice and lymph node counts for all experiments see **Supplementary Table S1**. Fixation of LNs was carried out at 4°C overnight in 3.2% paraformaldehyde (PFA) prepared from 32% aqueous solution EM grade (EMS Diasum, Cat. No. 15714 Hatfield, Pennsylvania, USA) in 1X phosphate buffered saline (PBS, Gibco™, Cat. No. 12549079, Thermo Fisher Scientific, Waltham, Massachusetts, USA). Subsequently, three washing steps in 1X PBS and embedding in 4% low-melting agarose followed. Organs were cut to a slice thickness of 100 µm with a Leica VT 1200 (Vibratome (Leica Biosystems, Wetzlar, Germany) with a cutting speed of 0.4 mm/s and a frequency of 1 mm. Blocking of nonspecific binding sites was achieved by incubating the tissue slices for 30 min in 5% normal goat or 5% normal donkey serum in PBS with 0.1% TritonX-100 (Carl Roth, Cat. No.: 9002-93-1, Karlsruhe, Germany) (0.1% PBST), respectively. Primary antibodies (**Table 2**) were incubated either for 2 hours at room temperature or overnight at 4°C. Subsequently, slides were washed 3x 10 min in 0.1% PBST and incubated with DAPI as a nuclear counterstain (Cell Signaling, Cat. No.: 4083S, Cambridge, UK) 1:1000 in PBS for 2 hours. For stainings using unconjugated (primary) antibodies, secondary antibodies (**Table 2**) were incubated together with DAPI. The slides were then washed 3x 10 min in 1X PBS and mounted with Vectashield Vibrance antifade mounting medium (Biozol, Cat.No. H-1700-10, Eching, Germany). Images were taken at a confocal laser-scanning microscope (cLSM), type Zeiss LSM-980 with Airyscan 2 (Zeiss Microscopy, Oberkochen, Germany).

**Table 2:** Primary and secondary antibodies with corresponding dilutions used for immunohistochemical staining.

Primary antibody	Clone	Lot no.	Supplier	Catalog no.	Dilution
CD45R (B220)	RA3-6B2	2785615	Invitrogen/eBioscience	14-0452-82	1:200
CD3	SP7	Z605-1	DCS	C1597C002	1:200
CD11c	N418	2471984	Invitrogen/eBioscience	14-0114-81	1:200
CD21/35	7E9	34907M1122	Invitrogen	MA5-49032	1:200
CD27	LG.3A10	2072708	Invitrogen/eBioscience	14-0272-82	1:100
CD40	HM40-3	2785615	Invitrogen/eBioscience	14-0402-82	1:100
CD80	16-10A1	AB_465132	Invitrogen/eBioscience	11-0801-81	1:200
CD117/c-KIT	poly	IEO0323121	Novus Biologicals	AF1356-SP	1:200
CD138	poly	YC368784	Invitrogen	36-2900	1:200
CXCR5/CD185	JB11-40	GR3342620-3	Invitrogen	MA5-41258	1:100
IgM	II/41	2727307	Invitrogen/eBioscience	11-5790-81	1:200
IgD	11-26c (11-26)	2691683	Invitrogen/eBioscience	11-5993-82	1:200
IgA	mA-6E1	2608932	Invitrogen/eBioscience	11-4204-82	1:200
IgG	poly	17	CellSignaling	4410S	1:1000
KI-67	SP6	1001370-3	Abcam	ab281847	1:50
LYVE-1	poly	1053718-2	Abcam	ab14917	1:100
MHC-II	M5/114.15.2	2297223	Invitrogen/eBioscience	14-5321-82	1:800
PNAd-647	MECA-79R	D104935	Novus Biologicals	NBP2-78792AF647	1:200
Vimentin	poly/ EPR3776	P1/ 1006594-53	Invitrogen/ Abcam	PA5-142829/ab92547	1:100
Secondary Antibody	Clone	Lot No.	Supplier	Catalog No.	Dilution
Donkey anti goat, DyLight^®^ 405	poly	46386	Novus	NBP1-72853	1:500
Donkey anti goat, Alexa Fluor™ 488	poly	GR3401020-2	Abcam	ab150129	1:1000
Donkey anti rabbit, DyLight^®^ 405	poly	D158450	Novus	NBP1-75286V	1:500
Donkey anti rabbit, Alexa Fluor™ 647	poly	GR3427773-1	Abcam	ab150063	1:1000
Goat anti armenian hamster, Alexa Fluor™ 488	poly	2789891	Invitrogen	A78963	1:1000
Goat anti rabbit, CF™ 488A	poly	23C0130	Sigma-Aldrich	4412	1:1000
Goat anti rabbit, Alexa Fluor™ 647	poly	27	CellSignalling	4414	1:1000
Goat anti rat, Alexa Fluor™ 488	poly	GR310150-2	Abcam	ab150157	1:5000
Goat anti rat, DyLight^®^ 550	poly	GR324582-3	Abcam	ab96888	1:1000

For IgM staining, LNs were frozen in liquid nitrogen and subsequently embedded in tissue preserving TissueTek^®^ O.C.T.™. LNs were then cut into 15 µm thin slices using a Leica Cryostat CM 1950 and placed on a glass slide. Slices were fixed with -20°C acetone for seven minutes. After drying for 5 minutes, the tissue was subsequently washed in 1X PBS and incubated with the IgM antibody for 1 h in a moist, dark chamber. The slices were then washed with 1X PBS and mounted with Vectashield Vibrance antifade mounting medium (Biozol, Cat.No. H-1700-10, Eching, Germany) and subsequently imaged with the cLSM.

### Synchrotron radiation-based microcomputed tomography

For a visual schematic step-by-step workflow of our preparation, acquisition, processing, analysis, and visualization pipeline, see our previous work by Schwarzenberg et al. (29). Here, mandibular, mesenteric and popliteal LNs were dissected from a 17-week-old and a 73-week-old mouse, respectively, by removing the surrounding tissue generously to preserve vascular structures. LNs were fixed in 4% PFA in PBS either 2 h at room temperature or at 4°C overnight. Subsequently, LNs were immersed for at least 1 hour in 70% EtOH and further dehydrated in a graded series of 2-Propanol. The organs were further stained with lead-hematein according to Müller et al. (59) with the modification that the acidic fixation step was omitted and the samples were directly incubated in a 1:1 mixture of working solution A and working solution B for approximately 48 h. From each pair, one LN was designated for histological analysis while the other LN was directly embedded under vacuum (-0.08 MPa) in Hard Plus 812 resin (EMS Diasum, Cat. No. 14115, Hatfield, Pennsylvania, USA) to enable subsequent ultrastructural analysis using scanning electron microscopy (SEM). All samples were mounted in polyimide (kapton) tubes to optimize scanning conditions due to their extremely thin tube wall of 0.075 mm, glued to sample stubs using UV hardening resin and sealed using 3D-printed plugs. The scanning setup was incorporated at the micro-tomography end-station at the imaging beamline P05 at PETRA III/DESY operated by Helmholtz-Zentrum Hereon (Geesthacht, Germany) (60, 61). To provide a monochromatic beam, an undulator source together with a double crystal monochromator were used. The detection system consists of a Ximea CB500MG camera with a CMOSIS CMV50000 sensor. The field of view covered by the beam within the five-fold magnification setup was about 7 mm in width and 3 mm in height. Samples were scanned at 20 keV with a 2D Talbot array illuminator setup (62–65) according to Schwarzenberg et al. (29). Phase retrieval was computed using the “Unified Modulated Pattern Analysis” (66, 67), after which the 2D differential phase was integrated and reconstructed *via* filtered back projection. The resulting image stacks featured an effective pixel size of 1.8 µm in an isotropic resolution after a two-fold binning. The detailed setup parameters were adjusted, and scanning of the biological samples was performed according to Riedel et al. (64, 68).

### Semi-automated segmentation with AI deep learning algorithms

Acquired synchrotron radiation-based microcomputed tomography (SRμCT) datasets comprised large 16-bit image stacks, which required preprocessing using ImageJ/Fiji (69) to reduce data size, computational load and scanning artifacts. In detail, image stacks were cropped as tightly as possible without compromising structural information. Since resin-embedded scans displayed edge artifacts at the air-resin interface due to the proximity of LNs to the resin border, the contrast gradients were corrected using Gaussian blur background subtraction with a sigma value of 300 - 500, followed by enhancement through an Unsharp Mask filter with 1.5 px and 0.4 - 0.6 weight as parameters. Then, the image stacks were converted to 8-bit and saved as JPEG files without compression. Analysis, segmentation, and visualization were performed using the Dragonfly software (Version 2022.2, build 1367 for Windows; Comet Technologies Canada Inc., Montreal, Canada). To optimize the datasets for deep learning, three preprocessing steps were applied: First, histograms were clipped to the relevant grayscale range corresponding to LN structures. Second, intensity values were normalized to a range between 0.0 and 1.0. Third, the datasets were converted to float32 format to ensure that the model could access fine-grained intensity gradients. The segmentation wizard was utilized for training deep learning models in an iterative, semi-supervised manner. The applied training regime can be described as follows: Eleven classes were defined for segmentation including LN parenchyma, medullary sinuses, deep cortical unit, B cell follicles, subcapsular sinuses, nodules, fat pad, outer vascularization, venules, capillaries and salivary glands (for mandibular LNs) or muscle (for popliteal LNs). Segmentation classes were defined by a combination of SRµCT-derived grayscale intensities, tissue morphology and anatomical context. For example, medullary sinuses and blood vessels were distinguished by their lumen-like appearance, low electron density, and vessel diameter, while follicles and nodules were annotated based on high density and spherical shape. The manual annotation of initial training frames as well as the semi-automatic segmentation was guided and cross-validated by established anatomical landmarks, histology and IHC to ensure biological plausibility of each class. Three entire frames were manually segmented as initial input data for early model training using the U-NET++ convolutional neural network architecture, and a fourth blank frame was reserved for monitoring the model performance during training. The following parameters yielded the best results: (i) setting the U-NET to 2.5D mode with three reference slices, (ii) increasing the patch size to 128, (iii) enhancing data augmentation to 12–15 transformations, and (iv) adding a Gaussian noise at a level of 0.2-0.3. The trained model was then used to predict 5–10 further frames, which were manually corrected to refine the model as much as possible. The finalized model was subsequently used for automatic segmentation of the entire image stack by interpolating segmentations along the x-, y-, and z-axes to create a 3D reconstruction. The resulting volumes underwent a final manual polishing by removing isles, smoothing, additional segmentation and subdivision of the outer vascularization into arteries, veins, as well as afferent and efferent lymphatics.

### Scanning electron microscopy

Excessive resin on the sides of the sample block containing no tissue has been removed with a razor blade. The tip of the block was used as a reference point to determine the distance to the first region of interest in correlation with the SRµCT data.

The trimmed sample block was mounted on a microtome (Leica Microsystems, Wetzlar, Germany; UC7), equipped with a Diamond knife (Diatome Ltd., Nidal, Switzerland, Cat.No.: DH4560), for cutting semi-thin sections of 200 nm thickness. Consecutive sections floating on the water bath were collected on a piece of silicon wafer (Ted Pella Inc., Redding, California, USA; Cat.No.: 16006) which has been pre-treated by glow discharge (Ted Pella Inc. easyGlow) to render the surface hydrophilic. The dried sections on the wafer were post-stained for 15 min in a 2% aqueous uranyl-acetate solution at room temperature in the absence of light followed by extensive washing with deionized water and repeated drying. To improve the conductivity of the section surface, a 2 nm carbon layer was deposited by carbon thread evaporation in high vacuum (Leica Microsystems, Wetzlar, Germany; ACE600).

Image acquisition was carried out on a Field Emission Scanning Electron Microscope (FESEM, Carl Zeiss AG, Oberkochen, Germany; Crossbeam 450) with an energy selective backscattered electron detector. Software automatization (v 5.2.2.85; Atlas Carl Zeiss AG, Oberkochen, Germany) for overview and detail images was used with the following parameters: accelerating voltage, 1.5 kV; probe current, 4 nA; working distance, 5.1 mm; ESB grid voltage, 500 V; pixel spacing, 200 nm (overview), 30 nm (detail); dwell time 25 µs.

### Histological analysis

After dissection, LNs were fixed by immersion in 4% PFA in PBS either 2 h at room temperature or at 4°C overnight. Subsequently, organs were dehydrated for at least 1 hour in 70% ethanol, further in a graded series of 2-Propanol and cleared using ROTI^®^Histol (Carl Roth, Cat. No. 6640.1) for two rounds of 1 hour each. Finally, tissue was infiltrated with 60°C warm Paraplast Plus paraffin for three rounds of 1 hour. The infiltrated samples were transferred into molds filled with Paraplast Plus paraffin and quickly cooled and solidified on ice. Paraffin blocks were trimmed and sectioned at 4 μm thickness using a Thermo Scientific HM 355S automatic microtome (Thermo Fisher Scientific, Waltham, Massachusetts, USA). Sections were mounted on microscope slides by floating on a warm water bath and dried overnight at 37°C. Staining was performed using Masson-Goldner Trichrome according to the standard protocol by Morphisto, with the following modifications: slides were incubated at 65°C for 20 minutes prior to deparaffinization through a descending alcohol series. Weigert’s iron haematoxylin was applied under warm tap water for 15 minutes. After washing for 8 minutes, the acid dye mixture was applied for 4 minutes. For this, the standard acid fuchsin-ponceau-azophloxin solution was substituted with brilliant crocein-acid fuchsin from the Movat pentachrome stain (which incorporates Verhoeff’s elastin staining) for its improved staining of murine erythrocytes. Samples were rinsed with 4% acetic acid for 30 s, incubated in prewarmed (60°C) phosphomolybdic acid-Orange G solution for 30 minutes, and rinsed again with 4% acetic acid for 30 s. Light Green Goldner III solution was applied for 12 minutes, followed by a brief rinse with isopropanol and then with 4% acetic acid for 2 minutes. Subsequent dehydration was conducted using an ascending alcohol series. A final clearing was performed using ROTI^®^Histol for two rounds of 5 minutes each, followed by the application of Roti^®^Histokitt mounting medium (Carl Roth, Cat. No. 6638.1). Coverslips were carefully added, and slides were allowed to harden for 12–24 hours. Imaging was performed using Zeiss Axio Imager m2M (Zeiss Microscopy, Oberkochen, Germany).

### SEM and SRµCT co-registration

For registration of the SEM images to the corresponding SRµCT reconstructed datasets, we employed a Python-based 2D–3D registration pipeline (70) which finds and fits the appropriate virtual tomographic slice plane to an input 2D image. The pipeline optimizes alignment iteratively by minimizing the mutual information metric through affine transformations, including rigid 3D transformations of the CT volume, irregular scaling and additional local shear correction of the SEM image. This process is performed with progressively coarse-to-fine sampling of the data arrays and decreasing parameter step sizes. As the pipeline requires effective threshold-based binarization of the datasets, we first performed a flat-field correction and retiling of the overview SEM image. Individual ESB image tiles were corrected by division with a gaussian-filtered feature-empty tile. The ImageJ stitching plugin (71) was then used to retile the full panoramic/overview image based on approximate grid coordinates with additional overlap computation, and linear blending. The corrected SEM image was then inverted to match the CT contrast. The first registration pipeline output consists of the shear-corrected, downscaled overview SEM which can be overlaid onto the registered CT slice, sharing the resolution and coordinate system of the SRμCT dataset. The saved affine parameters were then re-applied for shear-correcting transformation of the original-sized overview SEM, with bilinear interpolation of the registered CT slice for upscaling, in order to overlay the images at the SEM resolution. Finally, co-registration of the high-magnification (zoom) SEM image was achieved by applying the previous shear correction at the higher resolution, followed by additional refinement through further 2D iterations of the registration pipeline.

### Mass spectrometric proteomics

Mandibular and subiliac LNs were harvested from five young (17 weeks old) and five old (45–50 weeks old) mice, with two mandibular and two subiliac LNs per mouse. Whole LNs were lysed through addition of 0.1 M triethylammonium bicarbonate buffer (TEAB, Thermo Fisher) with 1% w/w sodium deoxycholate (SDC, Sigma Aldrich) and manually homogenized using a TissueLyser (TissueLyser II, Qiagen). Subsequently, the homogenized samples were incubated at 95°C for 5 min and sonicated with 10 pulses at 30% power. Protein concentration was determined by bicinchoninic acid-assay (BCAassay) according to manufacturer`s instructions (Pierce TM BCA Protein Assay Kit, Thermo Fisher). 10 µg protein diluted in 25 µL buffer were used for tryptic digestion. Tryptic digestion was carried out in a 96-well LoBind plate (Eppendorf, Hamburg, Germany) using a semi-automated setup on an Andrew+ Pipetting Robot (Waters, Milford, USA). Disulfide bonds were reduced with 10 mM dithiothreitol for 30 min at 56°C while shaking at 800 rpm, followed by alkylation with 20 mM iodoacetamide for 30 min at 37°C. Carboxylate-modified magnetic E3 and E7 speed beads (Cytvia Sera-Mag™, Marlborough, USA) were used in a 1:1 ratio, suspended in LC-MS grade water, and added to the samples at a 10:1 beads-to-protein ratio, based on the SP3 protocol workflow (72). Proteins binding was performed in 50% acetonitrile (ACN) while shaking at 600 rpm for 18 min. The magnetic beads were then washed twice with 80% ethanol and 100% ACN. Proteins were digested overnight at 37°C in 100 mM ammonium bicarbonate (AmBiCa) with sequencing-grade trypsin (Promega) at an enzyme-to-protein ratio of 1:100, shaking at 500 rpm. To inactivate trypsin, trifluoroacetic acid (TFA) was added to a final concentration of 1% and shaken for 5 minutes at 500 rpm. The resulting supernatant containing tryptic peptides was transferred to a new 96-well LoBind plate and prepared for subsequent LC-MS/MS analysis.

Differential quantitative proteomics measurement was performed using a quadrupole-orbitrap hybrid mass spectrometer (Exploris 480, Thermo Fisher Scientific) coupled to a nanoUPLC system (Vanquish TM neo UPLC system, Thermo Fisher Scientific). 1 μL of each peptide sample was injected into the chromatographic system via an autosampler. Peptide separation was achieved using a two-buffer system (buffer A: 0.1% FA in H_2_O; buffer B: 0.1% FA in ACN). Samples were first purified and desalted on a reversed-phase trap column (100 µm × 20 mm, 100 Å pore size, 5 µm particle size, C18, NanoViper, Thermo Fisher), and subsequently transferred to a 50 cm reversed-phase analytical column (μPAC™ Neo, 75 μm × 500 mm, 100 Å pore size, 2.5 μm pillar diameter; Thermo Fisher Scientific) for separation.

Chromatographic separation was performed using an 80-minute method with a linear gradient from 2% to 45% buffer B over 67 minutes. Peptides were ionized using a nano-electrospray ionization (nano-ESI) source with a spray voltage of 1,800 V. The ionized peptides were analyzed in data-independent acquisition (DIA) mode. For each MS1 scan, ions were accumulated for up to 240 milliseconds or until a target of 3 × 10^6^ ions (AGC target) was reached. MS1 scans were acquired using Fourier transform-based mass analysis in the Orbitrap within a mass range of 400–1400 m/z and a resolution of 120,000 at m/z 200. Within a precursor mass range of 380–980 m/z, DIA fragmentation was carried out using 12 m/z isolation windows with 1 m/z overlap, applying a normalized collision energy of 28% via higher-energy collisional dissociation (HCD). MS2 scans were acquired using the Orbitrap analyzer over a mass range of 350–2000 m/z with a resolution of 30,000, using a maximum injection time of 54 ms or until reaching an AGC target of 2 × 10^6^.

LC-MS/MS data were searched with the CHIMERYS DIA algorithm integrated into the Proteome Discoverer software (v3.1.0.638, Thermo Fisher Scientific) against a reviewed murine Swissprot database, obtained in June 2024, containing 17,163 entries, using Inferys 3.0 fragmentation as prediction model. Carbamidomethylation was set as a fixed modification for cysteine residues. Methionine oxidation was allowed as variable modification. Only peptides between 7 and 30 amino acids and with a maximum of one missing tryptic cleavage were considered. A strict cutoff (FDR < 0.01) was set for peptide identification, and the quantification was performed by CHIMERYS based on fragment ions. Normalization was applied to the total protein amount. Obtained protein abundances were log_2_-transformed. Data visualization was performed in R studio version 2024.12.0.467 (73) using the following packages: dplyr (74), tidyr (75), stats (76), ggplot2 (77), ggrepel (78), clusterProfiler (79), enrichplot (80), and org.Mm.eg.db (81). For each protein, mean abundance values were computed within four experimental groups: young subiliac, old subiliac, young mandibular, and old mandibular. These were used to calculate log_2_-fold changes (log_2_FC) across multiple comparisons: Mandibular vs. subiliac (mean, young, old), subiliac vs. mandibular (mean, young, old), Old vs. young within subiliac LNs, Old vs. young within mandibular LNs.

To assess the appropriate statistical test, each protein was first tested for normal distribution in each group using the Shapiro–Wilk test. Based on the result, either an unpaired t-test or a Wilcoxon rank-sum test was applied. This procedure was repeated independently for each comparison and protein. *P*-values were adjusted using Benjamini–Hochberg (FDR) correction to obtain *q*-values.

Proteins upregulated with age in both LN types were identified and removed from location-specific comparisons to isolate age-independent regional differences (**Supplementary Table S2**). The resulting filtered protein sets were used for volcano plot visualization based on *q*-values and GO term enrichment analysis based on *p*-values. Further, we performed marker-based enrichment using both single-sample GSEA (ssGSEA) and pre-ranked GSEA (fGSEA). Mouse MSigDB C8 cell-type sets together with curated B cell marker panels were intersected with the quantified proteome. The ssGSEA scores were computed on log_2_ protein abundances with the GSVA package (82) and visualized as row-wise z-scores. Group differences between mandibular and subiliac LNs as well as between ages were assessed using Wilcoxon tests with Benjamini-Hochberg (FDR) correction. For validation, differential t-statistics from a limma model were used as input for fGSEA (83), reporting normalized enrichment scores.

## Results

### Spherical cell aggregates inhabit the medullary region of murine mandibular lymph nodes at steady state

**Figure 1:**
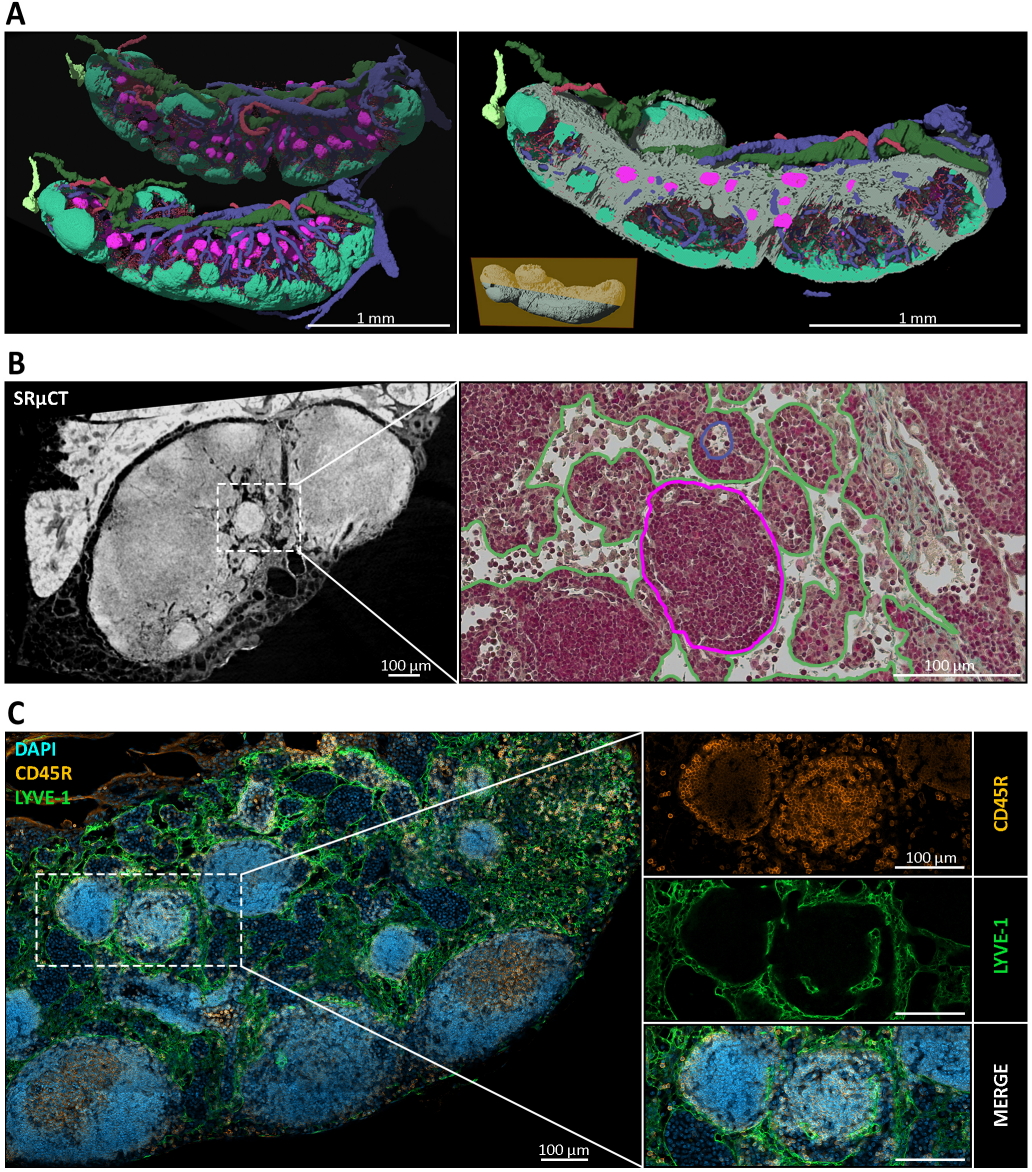
Spatial and structural characterization of medullary nodules in mandibular lymph nodes (LNs). **(A)** Three-dimensional (3D) reconstructions of mandibular LNs generated via synchrotron radiation-based micro-computed tomography (SRµCT), depicting distinct anatomical compartments. B cell follicles (mint green), medullary nodules (magenta), efferent lymphatic vessels (dark green), afferent lymphatic vessels (light green), venous blood vessels (blue), arterial blood vessels (red), and the surrounding medullary tissue (gray) are segmented and the deep cortical unit (DCU) is masked out to visualize their spatial relationships. Nodules appear as spherical to ovoid structures consistently embedded within the medullary compartment, clearly separate from conventional cortical follicles. An animated visualization of the model is available in the **Supplementary Files** (**Supplementary Video S3**) **(B)** Left: SRµCT cross-section of a mandibular LN reveals high-contrast spherical structures within the medulla. Right: correlative histological section stained with Masson-Goldner Trichrome. Nodules are visible as dense, spherical structures within the medulla (outlined in magenta), surrounded by looser medullary sinuses, with preserved tissue architecture (green outline), and accompanied by a medullary high endothelial venule (blue outline). **(C)** Immunofluorescence staining of mandibular LNs revealed the nodule architecture and cellular composition. CD45R (orange) marks B cells densely populating the nodules; LYVE-1 (green) labels lymphatic endothelial cells outlining the nodular periphery; and DAPI (blue) highlights nuclei. The merged panel confirms that nodules are composed of tightly packed CD45R^+^ B cells and are consistently encased by LYVE-1^+^ lymphatic structures, suggesting a potential integration into the sinusoidal network of the medulla.

In previous studies, we generated high-resolution three-dimensional (3D) image data of murine lymph nodes (LNs) using synchrotron radiation-based microcomputed tomography (SRµCT) (29). This technique enabled detailed structural and spatial analysis of the LN compartmentalization and vascular architecture. Follow-up experiments utilizing the same setup, but extending the target to LNs of multiple body positions, revealed spherical to ovoid, cell-dense structures within the medullary region of mandibular and accessory mandibular LNs (**Figure 1A**). Notably, these structures were observed in animals from multiple independent animal facilities (data not shown), indicating that their presence is not an artifact of housing conditions. To substantiate the high-quality but inherently low-throughput SRµCT datasets, we employed a multimodal approach by combining these datasets with correlative histology and scanning electron microscopy (SEM) for cellular context, and complementary immunohistochemistry (IHC) and proteomic profiling for molecular context. Further, the high-resolution SRµCT data clearly demonstrated that these formations were properly integrated into the medullary LN compartment (**Figure 1B**). Surrounding medullary sinuses remained intact, and the overall architecture of the mandibular LNs was preserved, implying that the presence or formation of these structures did not disrupt the native tissue organization. Subsequent multimodal analyses revealed similar structures in other mucosa-draining LNs. Based on their consistent morphology, we refer to these formations as *nodules*. The observed cell density of nodules appeared similar to that of B cell follicles. Naturally, we checked for the presence of B cells within nodules using immunohistochemistry staining against CD45R (formerly known as B220) – a standard B cell marker. Nodules proved to be CD45R^+^, identifying nodule inhabiting cells as B cells (**Figure 1C**). Although identification via conventional two-dimensional (2D) light microscopy proved more challenging than with 3D µCT imaging, we established a set of morphological and spatial criteria to reliably define nodules. These include: (i) exclusive localization within the medullary region, distinctly separate from B cell follicles and the paracortical zone; (ii) spherical to ovoid shape; (iii) a mean Feret diameter of 90 µm ± 27 µm, corresponding to an estimated true average of ~128 µm ± 38 µm after accounting for ~30% tissue shrinkage due to fixation, dehydration and resin embedding; and (iv) encapsulation by a PECAM-1^+^ and LYVE-1^+^ cell layer (**Figure 1C**), likely lymphatic endothelial cells, demarcating the nodule from surrounding tissue (84, 85).

**Figure 2:**
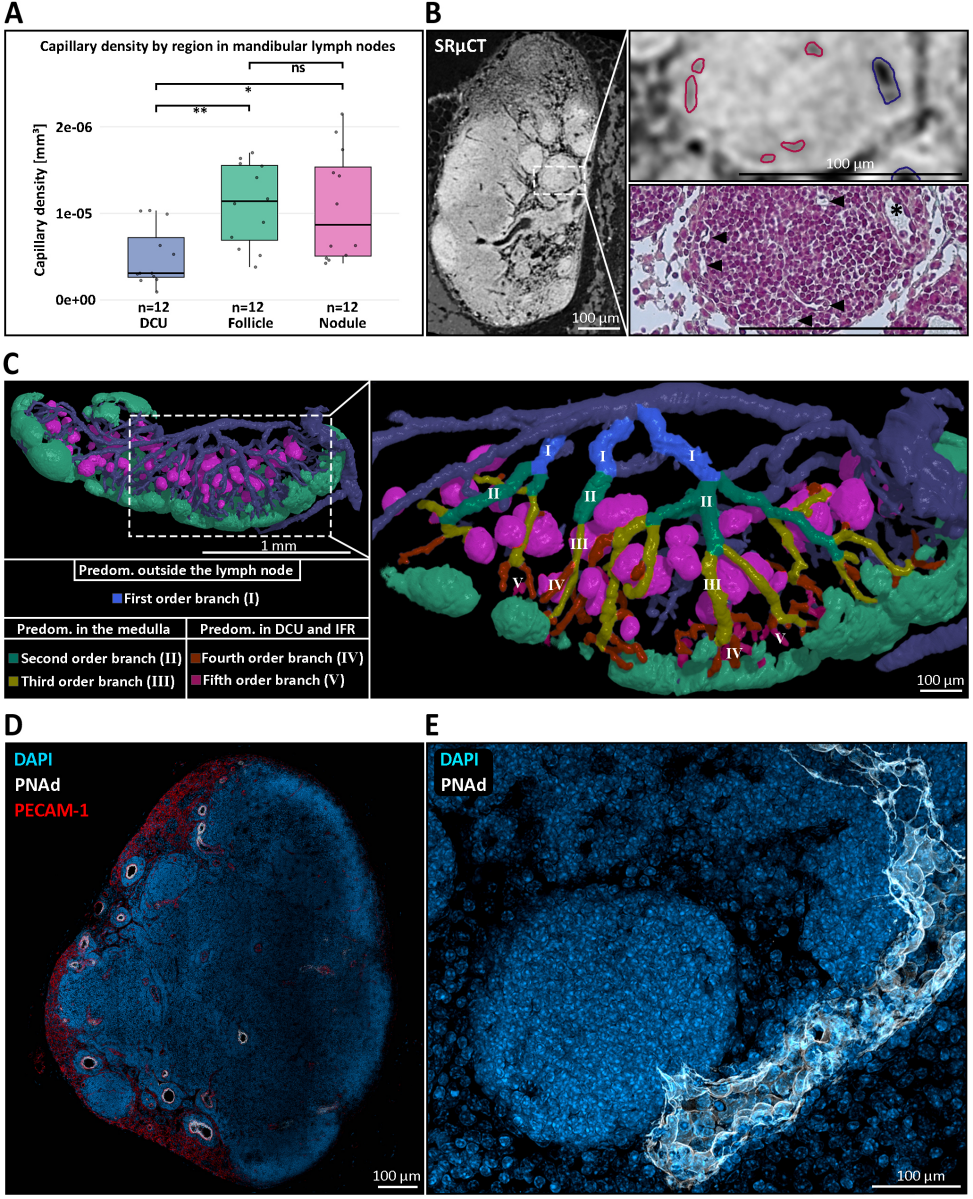
The blood vascular architecture within and around medullary nodules of mandibular lymph nodes (LNs). **(A)** Quantification of capillary density across distinct anatomical regions of the mandibular LN. Capillary density was significantly higher in B cell follicles (green box) and medullary nodules (magenta box) compared to the deep cortical unit (DCU) (blue box), with nodules showing a similar capillarization to follicles. Statistical significance was assessed using the non-parametric Wilcoxon test. **P ≤ 0.01, *P ≤ 0.05, ns=not significant, P>0.05. **(B)** Correlative imaging of vascular architecture. Left: high-resolution synchrotron radiation-based micro-CT (SRµCT) scan highlighting internal LN structure. Top right: high endothelial venule (blue outline) and capillaries (red outlines) were identified based on vessel morphology. Bottom right: correlative Masson-Goldner Trichrome-stained histological section confirms accurate vessel segmentation and showcases the medullary nodule capillarization (black arrowheads) and the heightened venular endothelium (asterisk). **(C)** Three-dimensional reconstruction of the venous branching hierarchy in the mandibular LN. Left: overview of nodules (magenta), follicles (mint green), and the venous blood vascular system. Right: magnified rendering of a reconstructed branching hierarchy with the posterior LN half being masked out for enhanced visibility. The branching pattern illustrates how nodules are primarily embedded in second- and third-order venous branches (green, yellow), whereas higher-order venules (IV, orange –V, purple) extend toward the deep cortical unit (DCU) and interfollicular regions (IFRs). This spatial arrangement highlights the restriction of nodules to the medullary compartment and suggests a close association between nodules and medullary venules. **(D)** Immunofluorescent staining of a mandibular LN showing peripheral node addressin (PNAd, white) expression within medullary HEVs, co-stained with DAPI (blue) and PECAM-1 (CD31, red). PNAd^+^ HEVs are observed outside the conventional paracortical zone, localizing in the medullary region, a deviation from canonical LN vascular architecture. **(E)** High-resolution confocal image of a single medullary nodule, illustrated by the densely packed lymphocytes through nuclear DAPI staining (blue) and the direct drainage of that nodule by a PNAd^+^ HEV (white), indicating potential lymphocyte entry sites into the nodule.

Three-dimensional reconstructions from µCT imaging provided two key insights into the vascular context of nodules. First, nodules displayed capillary densities comparable to those of B cell follicles. In our previous study we reported a 2.2-fold higher capillary density in B cell follicles compared to the deep cortical unit (DCU) (29). Current data using the same approach yielded a similar follicle-to-DCU ratio of 2.2, excluding one strong outlier (**Figure 2A**). Capillary densities measured in nodules were comparable to those measured in B cell follicles and resulted in a nodule-to-DCU ratio of 2.18, indicating similarly dense vascularization (**Figures 2A, B**) (**Supplementary Table S4**). Second, following first order venules located outside the LN, nodules were embedded within a venular network of second- and third-order venules, which connect to the DCU and interfollicular regions via fourth and fifth order venules (**Figure 2C**). Remarkably, high endothelial venules (HEVs), typically restricted to these fourth and fifth order branches (86), were observed within the medullary compartment of nodule-containing LNs through IHC staining against peripheral node addressin (PNAd) (**Figure 2D**). The HEV identity is further substantiated by the observed heightened endothelium in histological sections (**Figures 1B**, **2B**). This localization challenges the conventional view of HEV distribution located within the paracortex and the previously reported HEV localization directly at the B cell follicle border (29). Medullary HEVs with luminal PNAd expression in close proximity to nodules suggests a direct anatomical and potentially functional association between both structures (**Figure 2E**). Indeed, spatial proximity between medullary HEVs and nodules was consistently observed, supporting the hypothesis that these vessels may serve as entry points for lymphocytes accumulating in nodules. A systematic survey for PNAd^+^ vessels within the medulla across all murine LNs confirmed the presence of medullary HEVs in several mucosa-draining LNs. In contrast, no medullary HEVs were observed in typical skin-draining LNs – including subiliac, sciatic, popliteal, and axillary nodes – highlighting a body site-specific vascular feature potentially linked to nodule formation.

### Medullary nodules occur across lymph nodes from various anatomical sites, arise postnatally, and show a tendency to accumulate with age

Initial identification of nodules within the medullary regions of the mandibular and accessory mandibular LNs by SRµCT imaging prompted a broader investigation to determine whether these structures represent a general feature of the lymphatic system or are restricted to specific anatomical locations. As such nodules had not been previously described, we systematically examined LNs across different body regions. Using the nomenclature established by Van den Broeck et al. (58), we dissected 21 out of 22 anatomically defined LNs from four mice at an age of 15–20 weeks. Further, we deviated from this nomenclature for jejunal and colic LNs and summarized them under the term ‘mesenteric LN’ due to their clustered chain-like arrangement. Notably, the tracheobronchial LN could not be reliably dissected or identified due to its small size under steady-state conditions and inter-individual anatomical variability. Using IHC staining against CD45R we identified nodule-like structures within the medulla of mesenteric, superficial parotid, and lateral iliac LNs (**Supplementary Figure S5**). Notably, all nodule-containing LNs can be described as mucosa-draining LNs. Repeating the IHC analysis on LNs from three older mice (45–50 weeks) supported these findings. IHC data consistently indicated a significantly higher abundance of nodules in the mandibular and accessory mandibular LNs. These LNs typically contained multiple nodules per node, in contrast to other mucosa-draining LNs investigated, where nodules were markedly less frequent and often more difficult to detect.

**Figure 3:**
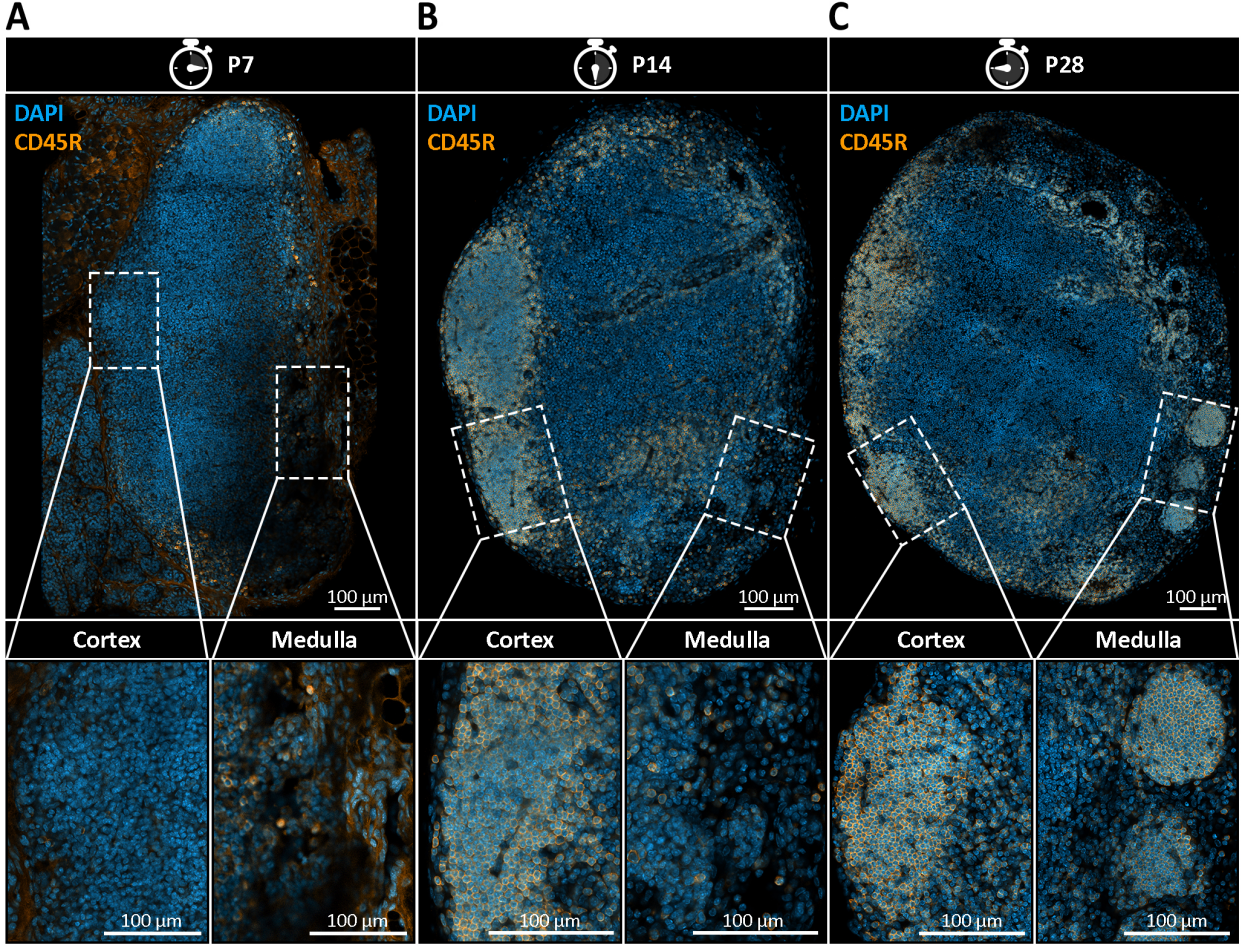
Postnatal emergence of medullary B cell nodules in mandibular lymph nodes (LNs). **(A–C)** Immunohistochemical staining of mandibular LN sections from postnatal day 7 (P7), day 14 (P14), and day 28 (P28) mice, using DAPI (blue) to label nuclei and CD45R (orange) to identify B cells. Representative images from four mice per time point with two lymph nodes per mouse are shown. **(A)** At P7, no distinct B cell follicles or medullary nodules are present. CD45R^+^ B cells are diffusely scattered throughout the LN, indicating early lymphoid colonization without compartmental organization. **(B)** By P14, the first CD45R^+^ B cell follicles begin to emerge within the cortical region, indicating the onset of follicular compartmentalization. However, no nodular B cell structures are observed within the medulla. **(C)** At P28, both cortical B cell follicles and CD45R^+^ spherical structures in the medullary region become clearly visible. These nodules are smaller, spatially distinct from follicles, and localized within the medullary compartment, indicating their delayed emergence relative to follicle development. Lower panels show magnified insets of cortical and medullary regions, emphasizing the spatial separation and morphological distinction between follicles and emerging nodules.

Understanding the temporal dynamics of nodule formation may provide key insights into their physiological relevance. To address this, we conducted a time-course analysis of mandibular and accessory mandibular LNs collected at postnatal weeks one, two, four, and eight, examining four mice per time point with two LNs per mouse (**Figure 3**). Nodular structures were identified by previously established morphological criteria and visualized using immunohistochemical staining with the B cell-specific marker CD45R which also facilitated identification of B cell follicles. Consistent with existing literature, B cell follicles were first reliably observed at two weeks of age, with initial B cell clustering detectable as early as one week of age (1) (**Figures 3A, B**). In contrast, nodules were not detectable at an age of two weeks and emerged until four weeks postnatally, indicating that their initial emergence occurs slightly delayed to the formation of B cell follicles between two and four weeks of age (**Figure 3C**).

**Figure 4:**
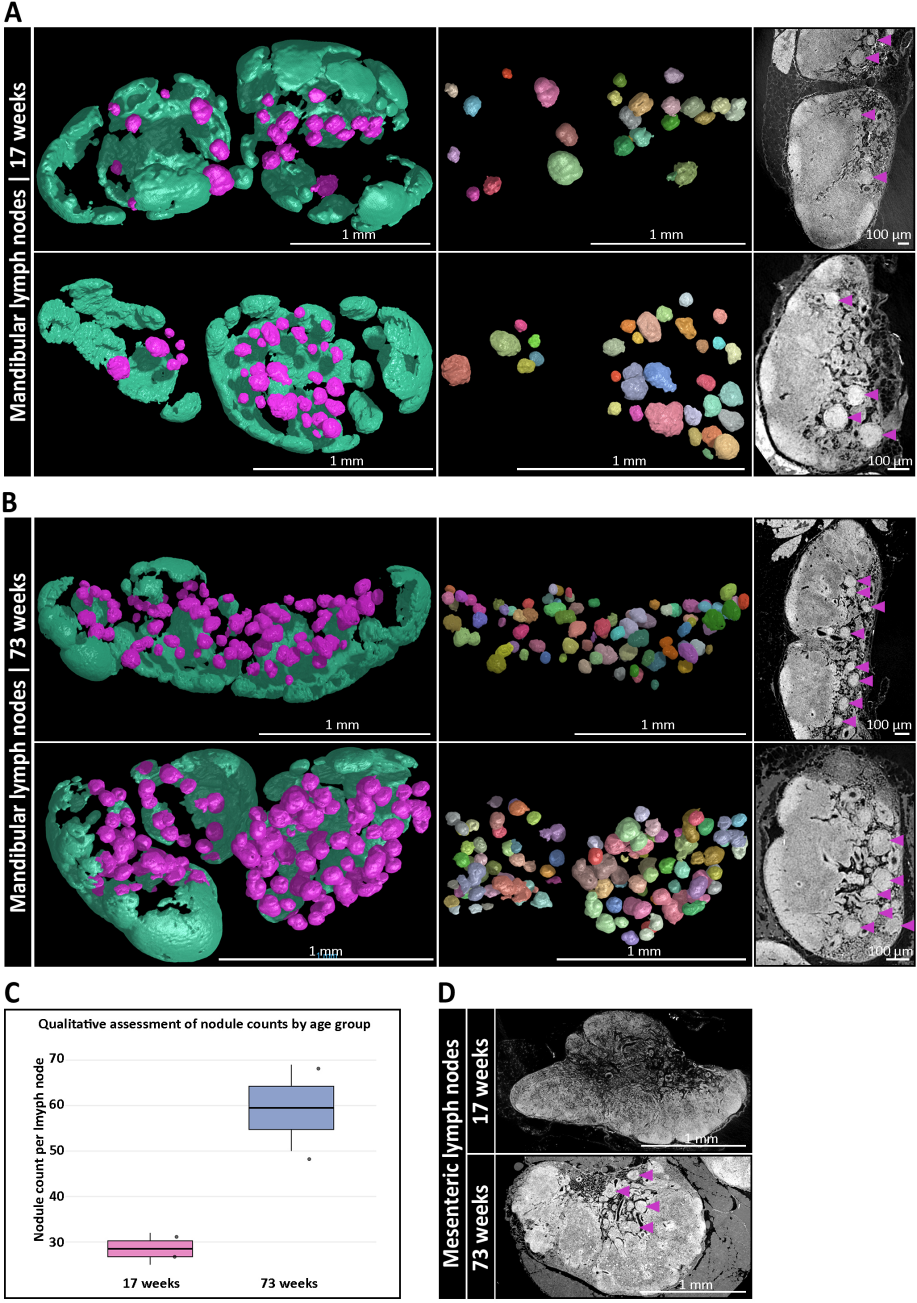
Age-dependent accumulation of medullary B cell nodules in mucosa-draining lymph nodes (LNs). **(A)** Three-dimensional (3D) reconstructions of mandibular LNs from a 17-week-old mouse, showing B cell follicles (mint green) and medullary nodules (magenta). Left: full LN volume rendering. Middle: isolated nodules through a multi-component analysis, each pseudo-colored to indicate individual structures. Right: corresponding synchrotron radiation-based µCT (SRµCT) slices confirm integration of nodules (magenta arrowheads) into the medullary compartment. **(B)** Equivalent 3D analysis of mandibular LNs from a 73-week-old mouse reveals a pronounced increase in the number and density of medullary nodules. Spatial organization and anatomical integration of nodules are preserved, while their abundance is significantly elevated compared to the younger mouse. **(C)** Qualitative assessment via high-resolution SRµCT analysis of nodule numbers per age group with two 17-week-old LNs vs. two 73-week-old LNs from one mouse, respectively. Boxplots show a descriptive twofold increase in the aged animal without inferential statistics due to the limited throughput of SRµCT. **(D)** SRµCT imaging of mesenteric LNs from 17-week-old and 73-week-old mice. Nodule-like structures (magenta arrowheads) are only present in aged animals. Anatomical localization remains consistent within the medullary regions.

To investigate potential age-related changes between LNs, we analyzed LNs from a young (17-weeks-old) and an aged mouse (73-week-old) using SRµCT. Since this experimental design was dictated by the throughput of SRµCT, the comparison was limited to one animal per age group with two LNs each and should be regarded as descriptive. In this dataset we observed an apparent increase in nodule abundance within mandibular and accessory mandibular LNs of the aged mouse, with a twofold increase compared to the younger counterpart. Specifically, the number of nodules increased from a mean of 28.5 in mandibular LNs of a young mouse to 59.5 in mandibular LNs of an old mouse (**Figures 4A–C**), corresponding to a mean of 1.9% and 4.7% of the total LN volume, respectively (**Supplementary Table S4**). Although this observation is currently restricted to mandibular and accessory mandibular LNs, a similar trend was also noted in aged mesenteric LNs (**Figure 4D**). However, due to the greater structural complexity of the mesenteric LN medulla, confident identification and quantification of nodules in this region proved challenging. Thus, while these findings suggest an age-related increase in nodule abundance, confirmation across larger cohorts using whole-mount 3D imaging is necessary to determine whether this is a generalizable feature of mucosa-draining, nodule-containing LNs.

### Mucosa-draining LNs exhibit enhanced immune readiness and resist age-associated stromal degeneration

**Figure 5:**
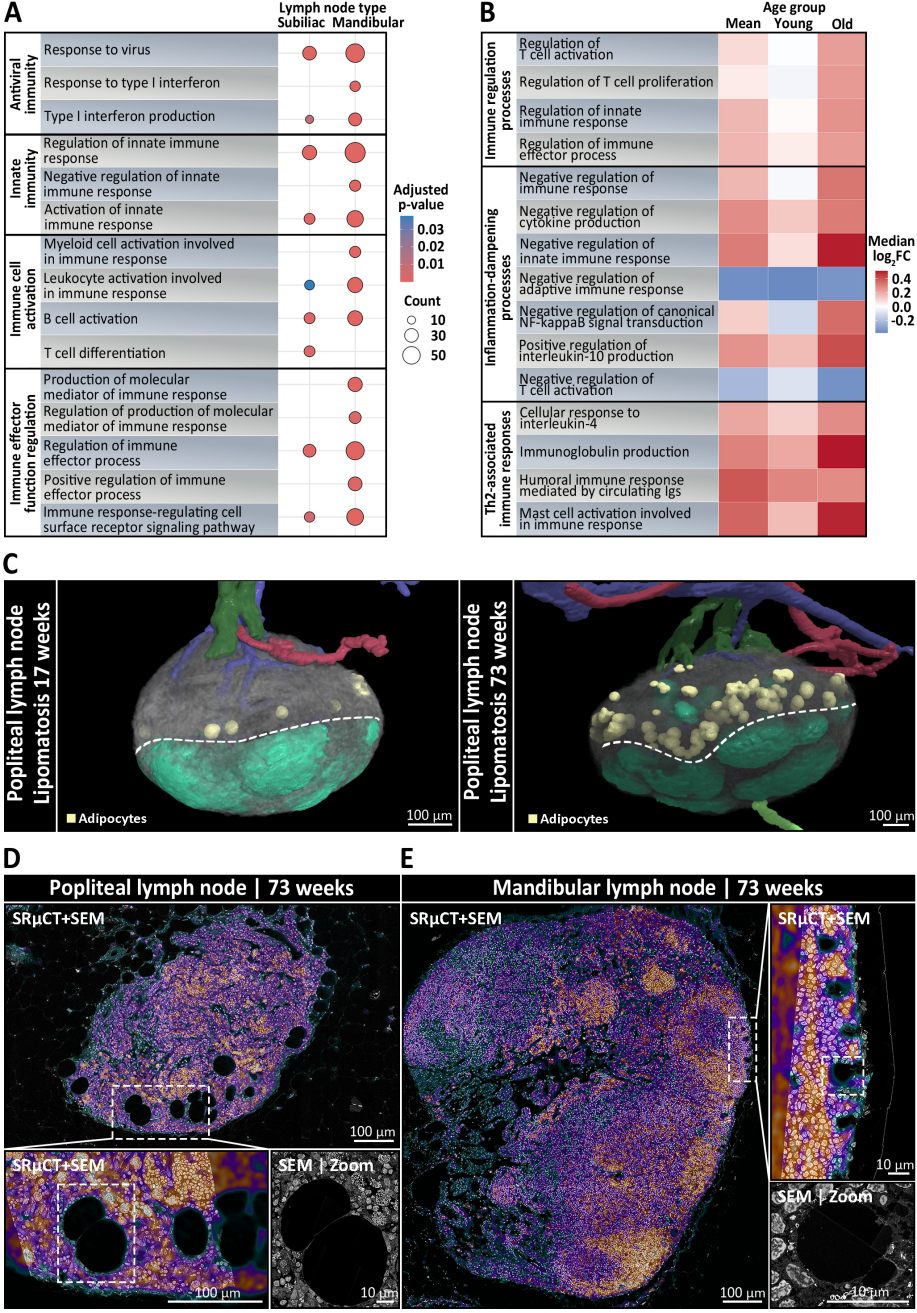
Immunological and structural differences between skin- and mucosa-draining lymph nodes (LNs) depending on anatomical localization and age. **(A, B)** Mandibular and subiliac LNs were harvested for bulk proteomics from five young (17 weeks old) and five old (45–50 weeks old) mice, with two mandibular and two subiliac LNs per mouse. **(A)** Dot plot showing the most diverse significantly enriched GO biological processes (Benjamini-Hochberg adjusted *p* < 0.05) between skin-draining subiliac LNs and mucosa-draining mandibular LNs, based on label-free quantitative mass spectrometry-based proteomic profiling. Circle size indicates the number of proteins associated with the respective GO term and color reflects statistical significance. Mandibular LNs are characterized by increased representation of pathways related to antiviral defense, innate immune activation, and lymphocyte priming, indicating heightened immune readiness, possibly due to continuous mucosal antigen exposure. **(B)** Heatmap displaying the relative log_2_-fold changes (mandibular vs. subiliac) in protein abundance of immune-regulatory biological processes across pooled mean, young, and old LN samples. Colors represent the median protein log2-fold changes for all proteins assigned to a given GO term, with red tones indicating increased abundance in mandibular LNs, white being neutral between the two, and blue tones displaying increased abundance in subiliac LNs. In young mice, mandibular LNs show only a mild skew toward immune regulatory, inflammation-dampening, and Th2-associated responses compared to skin-draining LNs. With age, these differences become increasingly pronounced, suggesting a progressive shift in the immunological landscape of mucosa-draining LNs, possibly reflecting cumulative effects of persistent low-grade mucosal antigen stimulation over time. **(C)** Three-dimensional rendering of skin-draining popliteal LNs with segmented arteries (red), veins (blue), afferent (light green) and efferent lymphatic vessels (dark green), adipocytes (yellow), and follicles (mint green) from synchrotron radiation-based µCT (SRµCT). Left: rendering shows that popliteal LNs of a young 17-week-old mouse were subject to very mild lipomatosis with only few adipocytes localized to the cortico-medullary border (dotted line). Right: rendering of an old 73-week-old mouse displays a strong increase in adipocytes, indicating pronounced lipomatosis. **(D, E)** Ultrastructural analysis using correlative SRµCT and scanning electron microscopy (SEM), with the SRµCT image overlaid on the SEM at 30% opacity. The correlative SRµCT image is displayed using the “Cool” lookup table from ImageJ, where black, green, and blue tones represent low-contrast areas such as adipocytes, vascular or sinusoidal cavities, while purple and red tones indicate high-contrast areas like leukocytes and stromal cells. **(D)** Cross section of a popliteal LN from a 73-week-old mouse. The inset highlights extensive lipomatosis characterized by large adipocyte accumulation within the medullary region. The SEM micrograph shows adipocytes displacing lymphoid tissue, indicating progressive loss of immune architecture in skin-draining nodes with age. **(E)** In contrast, a mandibular LN from a 73-week-old mouse displays preserved medullary integrity with rarely any visible signs of lipomatosis. SEM analysis reveals dense lymphocyte-rich areas and only few, considerably smaller adipocytes at the follicle-subcapsular-sinus-interface, suggesting active maintenance of lymphoid structure.

To delve deeper into site- and age-specific adaptations of mucosa- and skin-draining LNs, we performed quantitative proteomic profiling on whole mandibular and subiliac LNs, harvested from five young (17 weeks old) and five old (45–50 weeks old) mice with two mandibular and two subiliac LNs per mouse, resulting in 40 analyzed LNs in total. An approach of label-free mass spectrometry was followed by GO term enrichment to identify differentially represented biological processes. Among the most diverse and significantly enriched processes (Benjamini-Hochberg adjusted *p* < 0.05), mandibular LNs displayed increased representation of antiviral defense, innate immunity-related processes, immune cell activation and immune effector regulation (**Figure 5A**), suggesting enhanced immune readiness, likely due to continuous exposure to mucosal antigens. Comparative analysis of GO term-associated log_2_-fold changes between mandibular and subiliac LNs further revealed a mild tendency of mucosa-draining mandibular LNs toward inflammation-dampening and Th2-associated immune responses in young mice (**Figure 5B**). These differences, especially regarding immune regulatory processes, become more pronounced in aged mice, indicating a progressive shift in the immunological landscape of mucosa-draining LNs over time (**Figure 5B**). We further asked if the data allowed for an identification of a nodule-like B cell program and if it differed between the sites and with age. For this, we scored each sample using a single-sample gene set enrichment analysis (ssGSEA) using curated B cell panels together with MSigDB C8 cell-type gene sets and cross-validated with pre-ranked GSEA (fGSEA) on limma t-statistics (**Supplementary Figure S6**). While the analysis did not provide any evidence for a distinct nodule-specific B cell program in bulk proteomes, it consistently showed enrichment of B-lineage programs dominated by plasma/secretory signatures in mandibular LNs (FDR < 0.05) and this skew increased with age.

In addition to functional differences, correlative SRµCT and SEM analysis provided detailed insights into age- and body site-specific changes in LN composition. Popliteal LNs from a 73-week-old mouse exhibited classical forms of lipomatosis, characterized by the diffuse accumulations of large adipocytes located at the medullary-cortical border (**Figures 5C, D**) (**Supplementary Video S7**). These adipocytes were variable both in abundance and size, averaging ~100 adipocytes in popliteal LNs, with mean Feret diameters ranging from 40 µm to 90 µm after shrinkage correction, occupying up to 3% of the total LN volume (**Supplementary Table S4**). Interestingly, a mild degree of lipomatosis was already present in 17-week-old popliteal LNs with around 15 adipocytes, accounting for 0.26% of the LN volume (**Supplementary Video S8**). Conversely, nodule-bearing mandibular and mesenteric LNs of 73-week-old mice, featured virtually no visible signs of lipomatosis, which is particularly notable given their age (**Figure 5E**). Only a few, considerably smaller adipocytes were observed close to the follicles and the subcapsular sinus, while the medullary-cortical interface remained completely devoid of adipocytes (**Figure 5E**). These contrasting patterns may reflect region-specific differences in age-associated stromal remodeling across LNs.

### Medullary nodules host B cells with an unswitched, non-proliferative phenotype

**Figure 6:**
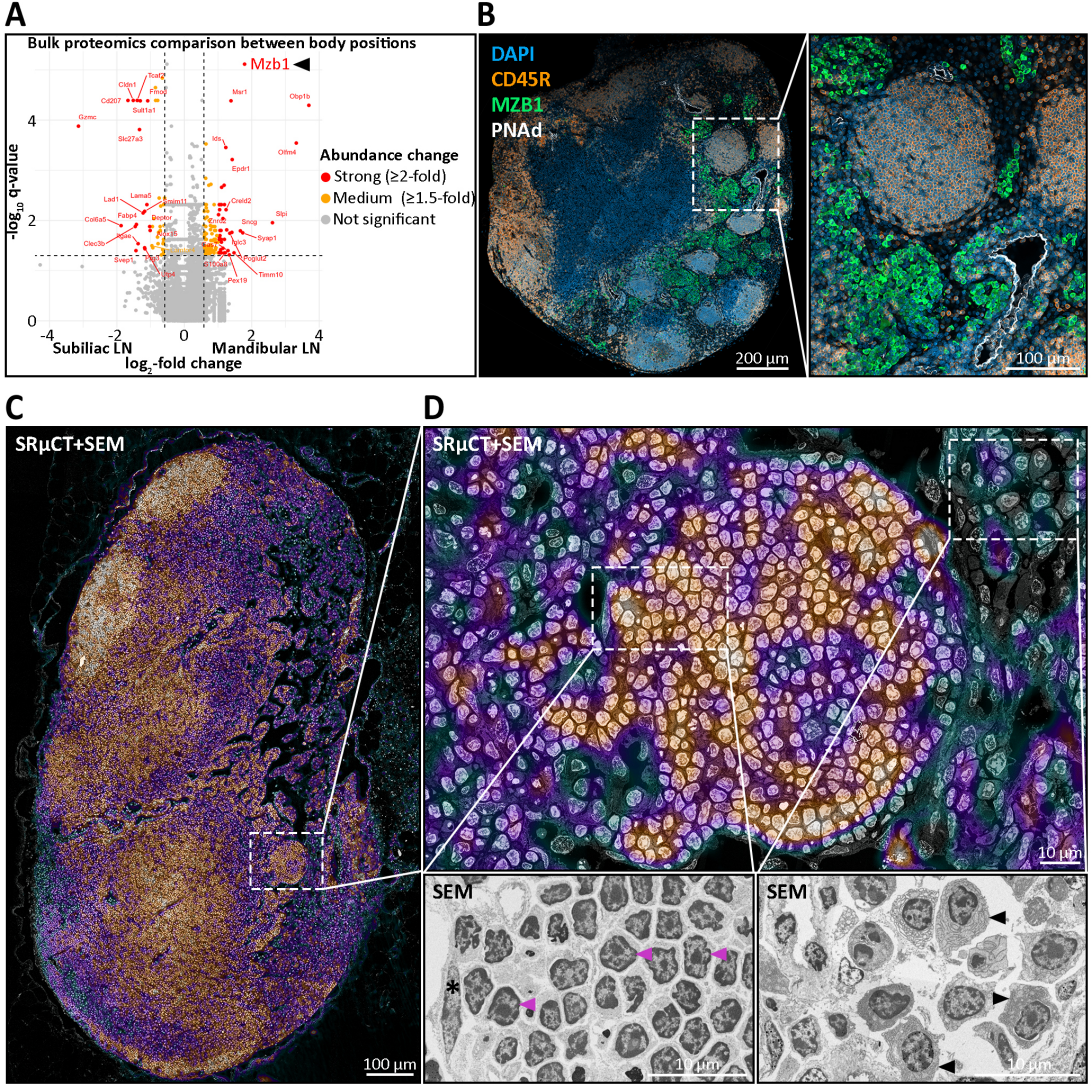
Molecular and ultrastructural characterization of medullary nodules in mandibular lymph nodes (LNs). **(A)** Bulk proteomics comparison of 20 mandibular versus 20 subiliac LNs pooled across age groups harvested from five young and five old mice, reveals significant differential abundances of proteins. Highlighted are strongly (≥2-fold, red), moderately (1.5–2-fold, orange), and non-significantly (gray) more abundant proteins. Notably, the B cell-associated chaperone MZB1, implicated in marginal zone and plasma cell biology, is markedly enriched in mandibular LNs. **(B)** Immunohistochemical (IHC) staining of a mandibular LN for DAPI (nuclei, blue), CD45R (B cells, yellow), MZB1 (green), and PNAd (high endothelial venules, white) shows distinct medullary nodules composed of CD45R^+^ B cells. Importantly, MZB1 expression is largely excluded from nodules, localizing instead to surrounding medullary regions. PNAd^+^ vessels are seen in close proximity to nodules. **(C, D)** Ultrastructural analysis using correlative synchrotron radiation-based µCT (SRµCT) and scanning electron microscopy (SEM), with the SRµCT image overlaid on the SEM at 30% opacity. The correlative SRµCT image is displayed using the “Cool” lookup table from ImageJ, where black, green, and blue tones represent low-contrast areas such as adipocytes, vascular or sinusoidal cavities, while purple and red tones indicate high-contrast areas like leukocytes and stromal cells. **(C)** Image of a mandibular LN section highlighting a medullary nodule and its anatomical context. The dense, spherical cellular aggregation is seamlessly integrated within the medullary region. **(D)** Ultrastructural morphology of nodular B cells. Cells within nodules show the classical resting lymphocyte morphology with dense heterochromatin radially distributed in a large, round nucleus and a slim cytoplasmic rim, excluding activated or effector cell identity. Lower insets display higher magnification. Left: representative lymphocytes with typical resting morphology (magenta arrowheads) and a nodule-enclosing lymphatic endothelial cell (asterisk). Right: plasma cells from the surrounding medullary tissue, characterized by elaborate endoplasmic reticulum (black arrowheads).

To delineate the cell composition of LN nodules, we employed a combined approach using mass spectrometry-based proteomics and IHC analysis with established lymphocyte and stromal cell markers (**Table 2**) (**Supplementary Table S2**). Notably, both LN types contained marginal zone B cell and B1 cell specific protein (MZB1) with mandibular LNs exhibiting a higher relative abundance of MZB1 showing a log_2_-fold change of 1.79, equating to a 3.46-fold increase (**Figure 6A**). MZB1 is highly abundant in plasma cells and can be found in the endoplasmic reticulum aiding in the assembly of the IgM-B cell receptor complex. Immunostaining confirmed the presence of abundant MZB1^+^ cells within mandibular LNs, predominantly localized in medullary cords. This finding is in line with the observed enrichment of plasma cell signatures in mandibular LNs (**Supplementary Figure S6**). In contrast, nodules were entirely devoid of MZB1^+^ cells (**Figure 6B**). Additionally, correlative SRµCT and SEM imaging of resin-embedded LN sections at a resolution of 30 nm revealed characteristic lymphocyte morphology, marked by a large nucleus, a thin cytoplasmic rim, and notably the absence of an elaborated endoplasmic reticulum, indicating that plasma cells were not present (**Figures 6C, D**). Supporting this, ultrastructural analysis confirmed that the average cell size of nodule-hosted B cells did not differ significantly from follicular B cells and averaged at 4.13 ± 0.55 µm, deviating from the native B cell size of 6-8 µm, likely due to fixation and dehydration (**Supplementary Table S4**).

**Figure 7:**
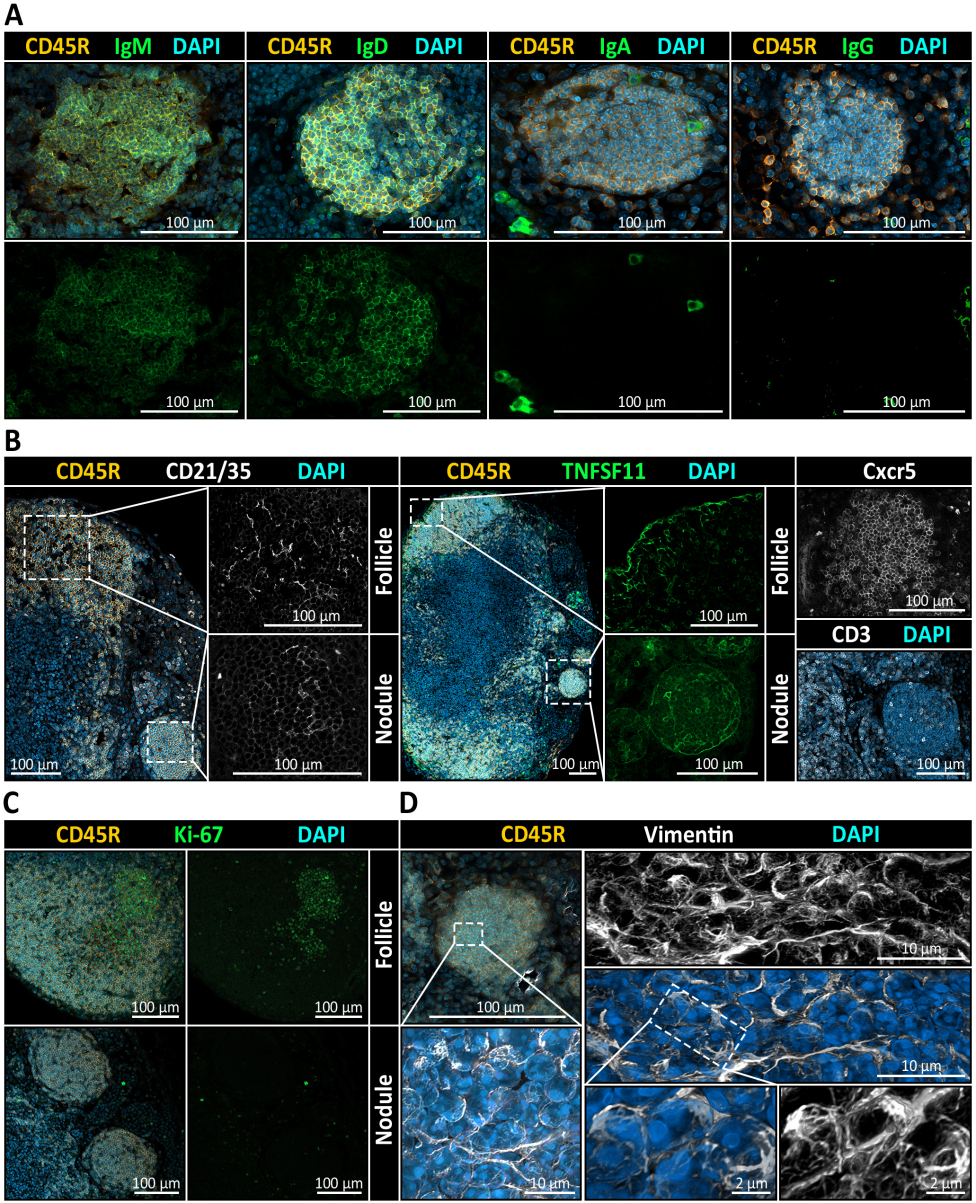
Immunophenotypic and stromal profiling of medullary B cell nodules in mandibular lymph nodes (LNs). **(A)** Immunohistochemistry (IHC) reveals the immunoglobulin expression pattern within CD45R^+^ nodules. Co-staining for IgM and IgD shows robust expression, while IgA and IgG are absent from nodular B cells. All sections are counterstained with DAPI to visualize nuclei. **(B)** Stromal and chemokine-related markers suggest follicle-like organization of nodules. Left: Co-staining for CD45R (B cells), CD21/35 (follicular dendritic cell marker), and DAPI reveals a clear CD21/35^+^ FDC network in cortical follicles and in medullary nodules. Middle: TNFSF11 (RANKL), a marker of marginal reticular cells (MRCs), is detectable at the border of follicles as well as delineating nodules. Right: CXCR5, a chemokine receptor mediating B cell migration toward CXCL13-enriched regions, is detected in both follicles and nodules, while CD3^+^ T cells are infrequently observed and only sparsely distributed within nodules. **(C)** Ki-67 is detected in cortical follicles but is virtually absent in nodules, indicating that nodular B cells are non-proliferative and lack germinal center activity. **(D)** Analysis of nodular B cells with Vimentin, a cytoskeletal marker associated with antigen-experienced B cells and stromal elements, displays as maximum intensity projection and as three-dimensional rendering a uniform and diffuse distribution, consistent with a quiescent, non-activated state of the B cells residing there.

Furthermore, nodular B cells expressed IgD and IgM (**Figure 7A**) as well as the chemokine receptor CXCR5, the latter indicating a potential for CXCL13-mediated migratory behavior typical of naïve B cells (**Figure 7B**). To assess the presence of CXCL13-producing cells associated with nodules, we performed immunohistochemical staining for CD21/35, a marker of follicular dendritic cells (FDCs), and TNFSF11 (also known as TRANCE), a marker of marginal reticular cells (MRCs). Both cell types are well-established as principal sources of CXCL13 within the LN microenvironment (87). Notably, TNFSF11 staining revealed a distinct signal encircling nodules, closely resembling the pattern observed at the borders of B cell follicles and a CD21/35^+^ network-like signal was detected within nodules (**Figure 7B**). This suggests that nodules may reside within a chemokine-guided microenvironment analogous to that of conventional B cell follicles. Also, CD3^+^ T cells were infrequently detected within nodules, representing only a minor subset of the cell population (**Figure 7B**). Despite their resemblance to B cell follicles, nodules lacked hallmarks of activation or proliferation. Stainings for activation and germinal center-associated markers KI-67 (**Figure 7C**), CD40 and CD80 (data not shown) were negative. Similarly, vimentin – typically polarized in activated B cells – was distributed evenly in nodule-resident B cells, consistent with a naïve phenotype (**Figure 7D**). Furthermore, plasma cell differentiation was excluded based on their characteristic morphology in SEM, and immunostaining for PD-L2 (data not shown) and for immunoglobulin isotypes IgA and IgG failed to detect class-switched memory B cells (**Figure 7A**). Taken together, these findings suggest that nodules share key architectural features with B cell follicles but remain in a quiescent, non-germinal center state.

## Discussion

### Discovery of medullary nodules as novel structures

To our knowledge, this is the first report of distinct B cell follicle-like nodules that are consistently situated in the medullary region of murine mucosa-draining lymph nodes (LNs). We were able to identify these spherical CD45R^+^, CXCR5^+^, IgD^+^, IgM^+^ B cell accumulations in high abundance in mandibular and accessory mandibular LNs and gathered evidence for their presence at multiple other anatomical sites, including mesenteric, superficial parotid, and lateral iliac LNs. The nodules always appeared to be seamlessly integrated into the medullary architecture with a LYVE-1^+^ layer, indicating potentially lymph endothelial cells (LEC), surrounding them (**Figure 1**). LECs are responsible for lining various lymphoid compartments including medullary sinuses. In addition to establishing a physical scaffold for antigen efflux and lymphocyte egress, they were shown to complement follicular dendritic cells by capturing and archiving antigens (88, 89), functioning as nonhematopoietic, nonprofessional antigen-presenting cells (90), and play a potential role in immune tolerance through presentation of PD-L1 (91, 92). The presence of a consistent LYVE-1^+^ layer around the nodules suggests a direct connection to the sinus networks. B cell survival is strongly dependent on chemokine signaling such as B cell activation factor (BAFF). BAFF is mainly produced by fibroblastic reticular cells (FRCs) and related cell types. In the medulla, specialized fibroblasts (medullary reticular cells) likewise provide such survival factors that support B lineage cells, particularly plasma cells, in that compartment (93). These findings suggest that B cell-dense medullary nodules could contribute to B cell homeostasis by positioning naïve B cells in proximity to stromal cells supplying survival signals. Furthermore, their positioning is of particular interest, since it allows for antigen surveillance and sampling. In fact, medullary sinuses are no mere drainage pipes but have proven to be immunologically active sites where antigen grabbing and presentation is still possible (94). Medullary dendritic cells (MDCs) were identified as essential mediators of humoral immunity by capturing lymph-borne influenza virus using the lectin receptor SIGN-R1 (95). While medullary sinus macrophages (MSMs) and MDCs are thought to be relatively sessile (96), the motility of virus-bearing MDCs increased by a fourfold and was directed toward FDC regions in a non-random manner (95). Since we also detected an CD21/35^+^ FDC-like network within the nodules (**Figure 7**), this raises the possibility of a similar migration toward nodules in case of viral infection. Further, while the precise functions remain unclear and require validation, this anatomical organization could represent a functional niche for nodular B cells that might be adapted for lymphocyte homeostasis, antigen handling and maintaining immunological quiescence. The functional adaptation of this putative niche could be explained by multiple properties of the LECs. First, one function could be guiding B cells to this structure. While high endothelial venules (HEVs) are the primary port of entry, guidance is driven by chemokines, which are generally not produced by blood endothelial cells (BECs) (97, 98). However, there is rising evidence that LECs in LNs play a crucial role in B cell homing through CXCL13 production (98). Second, while LECs are not known to present antigens to B cells directly, they might indirectly control B cell activation or quiescence by anatomical seclusion from cortical and paracortical regions, or by regulating antigen availability due to their antigen capture and archiving capabilities.

### Nodule microenvironment mirrors non-reactive B cell follicles

While LECs may contribute to initial CXCL13-mediated B cell recruitment, conventional B cell follicle formation relies primarily on follicular dendritic cells (FDCs) and marginal reticular cells (MRCs), which act as stationary producers of CXCL13 in the LN cortex (87). Although ectopically situated in the medulla, these nodules in fact exhibited a CD21/35^+^ network, which could resemble that of FDCs seen in B cell follicles. FDCs in follicles bind native antigen-antibody complexes via complement receptors and hold them for B cell surveillance over long durations (99). However, regarding B cell follicles, MRCs can translocate antigen from sinuses into the LN cortex, even in the absence of FDCs with naïve B cells transporting opsonized antigen from the sinus to FDCs (100). The observed TNFSF11^+^ (**Figure 7**) cells surrounding the nodules suggest the presence of MRC-like cells, which taken together would recapitulate the key stromal elements of follicular organization and could indicate a potential transportation capability for antigens carried inside lymph in medullary sinuses into nodules. Recent spatial transcriptomic analyses show B cells aggregate not only in follicles but also in the LN paracortex, co-localizing with reticular stromal cells that maintain homeostatic niches (101). However, we did not detect any clues that would hint toward proliferation or activation of nodular B cells. Specifically, nodules were completely devoid of germinal center (GC) activity and any typical proliferation and activation markers including KI-67, CD40, and CD80. Further, vimentin-positive fibers were homogeneously distributed, arguing against antigenic stimulation of the B cell receptor, which is generally accompanied by polarized vimentin signals (102). The nodular B cells are also likely not a result of a completed GC response, since they neither exhibited prominent rough endoplasmic reticulum (**Figure 6**), typical of antibody-producing plasma cells (103), nor did they express class-switched immunoglobulins (**Figure 7**). The absence of such cues however does not necessarily equate to an anergic state of the nodular B cells. While this would pose a possible explanation, naïve resting B cells typically have a low or undetectable expression of activation markers and are non-proliferative (104, 105). Additionally, only single CD3^+^ T cells were detected within nodules (**Figure 7**), and their medullary localization likely impedes efficient T cell contact. Given the follicle-like organization of these nodules, we next investigated vascular features and anatomical contexts that could explain their unusual medullary location.

### Specialized HEV-rich vasculature in nodule-bearing LNs and their site-specific dependence

Typically, HEVs are restricted to the T cell-rich zones and directly at the cortical-paracortical border closely associated with the B cell follicles of the LN. HEVs are marked by their high endothelium as well as their functional capability of lymphocyte homing through the expression of peripheral node addressin (PNAd) on the lumen side (24, 29, 86). The presence of PNAd^+^ vessels within the medullary regions of mucosa-draining LNs in this study was therefore unexpected (**Figure 2**), and these vessels have received only little attention in literature, where they are described as sparse and their roles poorly defined (106). Notably, medullary PNAd^+^ vessels have been described in brachial LNs during acute inflammation, where neutrophils were found to specifically localize to these vessels instead of the paracortical HEVs (107). However, their consistent presence in specific LNs under steady state conditions, as we report here, represents to our knowledge a novel finding. Recent studies have pointed out the transcriptional and molecular heterogeneity of high endothelial venules depending on the microenvironment, anatomical site, and inflammatory state of the organ (24, 25, 108). Peripheral lymph nodes mainly express PNAd, while mucosa-draining lymph nodes tend to co-express PNAd with the mucosal addressin cell adhesion molecule (MAdCAM-1) and Peyer’s patches often exclusively express MAdCAM-1 (26). Within this context, luminal PNAd remains the widely accepted marker for functional HEVs also in mucosal LNs, particularly when combined with morphological evidence of heightened endothelium. Still, thorough investigation of the heterogeneity of medullary HEVs might uncover valuable information on their formation, maintenance and response to inflammatory cues in future studies. Importantly, among other cell types like CD11c^+^ dendritic cells, B cells appear to play a critical role in orchestrating the formation and maintenance of the HEV network through lymphotoxin-β (LT-β) production and subsequent LT-β receptor (LT-βR) engagement to endothelial cells (109, 110). This signaling is required for PNAd and MAdCAM expression on HEVs to facilitate lymphocyte entry (111). Furthermore, LT-β was shown to be essential for normal cellularity of secondary lymphoid organs (SLOs), for the maintenance of FDC networks as well as for proper antigen sampling and presentation by antigen presenting cells (APCs) (112–114). Abe et al. (109) demonstrated that during immune challenge, antigen-draining LNs undergo structural remodeling of the medullary regions, a process dependent on plasma cells and non-cognate B cells. Their findings highlight LEC reorganization and optimization of a plasma cell niche during inflammation. Hence, their concept of B cell-driven medullary remodeling supports our data with the nodular B cells playing a central role in these specific LNs. The consistent presence of nodular B cell aggregates alongside PNAd^+^ medullary vessels raises the possibility that B cell-driven stromal remodeling sustains HEV structures within the medulla, which in turn promotes B cell homing to the nodules as a positive feedback mechanism. While our study focused on the vascular microenvironment, future work should include analysis of the reticular stromal network, as these may critically shape the architecture and stability of medullary nodules.

The anatomical dependence of nodule formation and the associated extensive HEV networks may provide critical insights into the factors driving their emergence. Numerically, most nodules were observed in mandibular and mesenteric LNs, which are also among the largest LNs in mice (115). These mucosa-draining LNs in particular operate under continuous antigenic exposure from commensal microbiota and environmental antigens including dietary antigens (47), resulting in a constant state of controlled, low-level immune activation, even in the absence of infection (44, 116, 117). We hypothesize that the abundance of nodules could be directly influenced by the strength and frequency of microbial and dietary antigen influx. Mandibular LNs might develop the highest nodule numbers due to direct and immediate exposure to oral antigens, whereas mesenteric LNs could exhibit fewer nodules as Peyer’s patches partly mitigate antigenic load before lymphatic drainage reaches them (118). Finally, other mucosa-draining LNs might display the lowest number of nodules because they are positioned further downstream, or because their respective mucosal sites host fewer commensals or encounter lower levels of antigenic stimulation (119). This hypothesis warrants further experimental investigation to elucidate the precise relationship between antigen exposure and nodule formation.

Nonetheless, chronic or repeated antigenic stimulation is widely recognized as a driving factor for HEV formation and remodeling of lymphoid tissue microarchitecture (120, 121). This can on the one hand be observed in non-lymphoid tissue, leading to the development of TLOs that exhibit ectopic HEVs (122, 123). On the other hand, evidence shows that constant antigenic stimulation through commensal microbiota strongly impacts LN development, where mesenteric LNs under germ-free (GF) conditions exhibit significantly less lymphocyte abundances, less developed follicles and significantly lower capabilities to respond to and clear infections (124–129). In line with this, mucosa-draining LNs display unique properties that differentiate them from skin-draining LNs. For instance, transcriptomic profiling indicates that mesenteric LNs display a cytokine environment that is skewed toward tolerogenic and inflammation-dampened responses through IL-4, IL-10, and TGFβ, which poises these LNs for T helper type 2 (Th2) and regulatory T cell (Treg) immunity. Keeping balance between tolerance and inflammation-induced immunity characterizes mucosa-draining LNs (130, 131). In contrast, skin-draining LNs are more quiescent at steady-state and predisposed toward T helper type 1 responses (132). Consistent with these observations, our bulk proteomic profiling revealed increased representation and higher protein abundances associated with immune readiness-related and immunoregulatory pathways in mucosa-draining mandibular LNs compared to skin-draining subiliac LNs, with a progressively greater divergence observed in aged mice. This may reflect the cumulative effects of persistent low-grade mucosal antigen stimulation with age, promoting adaptation toward immune tolerance and regulation. Our single-sample gene set enrichment analysis was not able to detect and specify the cell program of medullary-nodule-specific B cells using bulk proteomes. This could reflect signal dilution in whole-organ bulk proteomics as nodules represent only a small fraction of the lymph node microenvironment or could be due to the lack of missing nodule-like B cell reference signatures in currently available atlases. These limitations argue for compartment-directed approaches for example through laser-capture microdissection to enable nodule-focused insights in future studies.

In line with our proteomics data, our identification of B cell-rich medullary nodules within mucosa-draining LNs reveals a previously uncharacterized structural compartment that may also reflect a specialized adaptation to continuous mucosal antigen exposure. These nodules share striking features with the ectopic B cell clusters reported by Daniel et al. (133) in mediastinal LNs during chronic *Mycobacterium tuberculosis* infection. In both cases, B cells accumulate outside conventional follicular zones, are non-proliferative, and do not express typical activation or germinal center markers. However, while the ectopic clusters arise in response to infection and actively displace the fibroblastic reticular cell (FRC) network, contributing to impaired T cell activation, our described nodules appear under steady-state conditions and without overt disruption of LN architecture. This suggests that similar stromal and lymphocyte interactions may underlie both physiological and pathological LN remodeling, with context and inflammatory status determining the functional outcome. It remains to be determined whether these nodules serve a regulatory or antigen-sampling role in mucosal immunity, or if they could be co-opted into a dysfunctional state during chronic inflammation.

### Impact of aging on nodule formation and lymph node remodeling

Given that the anatomical distribution of nodules appears closely linked to chronic antigenic exposure, it is conceivable that sustained antigen-driven immune stimulation also influences the temporal dynamics of nodule formation, particularly in the context of aging. Indeed, our findings indicate that the formation of medullary B cell nodules is age-related, being absent in neonatal LNs and first appearing slightly later than primary follicles, around 2–4 weeks postnatally (**Figure 3**). This timing strongly suggests that nodule formation is closely tied to postnatal immune system maturation and increased antigen exposure. Consistent with this hypothesis, nodules arise shortly after the weaning period, when maternal nursing ends and pups start consuming solid food (134, 135). This dietary transition not only substantially elevates the intake of microbial and dietary antigens but also increases the likelihood of microlesions forming within the oral mucosa, further amplifying antigen influx to the mandibular LNs (136). The observed tendency of progressive increase in nodule numbers with advancing age further supports our hypothesis that these structures accumulate due to sustained, chronic antigenic stimulation throughout the lifespan. It was shown that chronic exposure like repeated antigenic stimulation (e.g. in allergy or autoimmunity) can cause persistent germinal centers or tertiary lymphoid structures in LNs (121, 137). Over time, nodules could either become active tertiary follicles or remain as quiescent repositories of memory/naïve B cells. In our SRµCT dataset we observed a higher number of medullary nodules in the aged compared with the young animal (17 weeks, 28.5 nodules vs. 73 weeks, 59.5 nodules), which would align with the idea that these structures accumulate as quiescent niches. However, because this comparison was limited to one mouse per age group, it remains a preliminary observation that requires future confirmation in larger cohorts across different LN locations. Notably, even when germinal centers eventually wane, antigen can remain deposited on stromal surfaces (138). This means a medullary nodule, formed during a chronic response, might continue to harbor antigen and memory B cells long after the active response, thereby serving as an archive for rapid reactivation. Conversely, in chronic tolerogenic exposure, such as constant food antigen influx to mandibular or mesenteric LNs, one might speculate that B cell nodules develop a tolerized phenotype, perhaps rich in regulatory cells or inhibitory stromal signals, though direct evidence for this is still emerging. Therefore, it would be insightful to investigate whether nodules form at all in GF mice or whether alternative, antigen-independent factors might drive their formation.

It has previously been established that aging impacts LNs at different anatomical sites distinctively, with peripheral skin-draining LNs being more susceptible to lipomatosis, which is the gradual replacement of lymphoid parenchyma by adipocytes, compared to mucosa-draining LNs (139). Our data aligns well with these observations, demonstrating substantial lipomatosis in popliteal and subiliac LNs, while notably highlighting a complete absence of this phenomenon in mandibular and mesenteric LNs (**Figure 5**). This disparity supports the “use-it-or-lose-it” concept, whereby continuous antigenic exposure in mucosa-draining LNs might actively maintain lymphoid structures, while relative antigenic quiescence in peripheral LNs could permit adipocyte infiltration. Specifically, we localized lipomatosis predominantly at the medullary-cortical interface of skin-draining LNs, a region particularly susceptible to adipogenic transdifferentiation of medullary reticular cells, compared to the more stable T cell zone reticular cells and FDCs (106). Importantly, adipocyte accumulation impairs LN functionality by disturbing HEV homeostasis, thus reducing lymphocyte entry, and obstructing lymphocyte egress and lymph flow due to disruption of medullary sinuses (106). This lipomatosis-driven impairment of LN function primarily results from reduced LT-β signaling, as sustained LT-βR activation prevents stromal cells from differentiating into adipocytes (140). Considering that B cells are significant sources of LT-β (141, 142) and that our nodule-bearing LNs showed virtually no lipomatosis, we propose that these nodules could preserve structural and functional integrity of mucosa-draining LNs in aged mice through persistent LT-β signaling.

Interestingly, age-associated changes in HEVs differ between human and mouse LNs. Human LNs typically exhibit substantial age-related reduction of paracortical HEVs (143), whereas mouse HEVs display relatively mild alterations, including moderate thinning and impaired T cell diapedesis (144). In this context, it would be interesting to investigate whether mucosa-draining murine LNs might even show an increase in overall HEV proportion with age, driven by the medullary HEVs described in this study, or whether HEV density remains unaffected by aging.

### Putative nodular B cell subtypes

We identified CD45R^+^, CXCR5^+^, IgD^+^, and IgM^+^ nodular B cells, embedded within a TNFSF11^+^ signal and supported by a CD21/35^+^ scaffold (**Figure 7**). Despite their quiescent phenotype, these nodules are unlikely to be “function-less” B cell deposits or pathological hyperplasias. Nodule frequencies double with age, arguing for a regulated, age-dependent process rather than random overgrowth. We propose that, despite the lack of evidence for activation or proliferation, nodular B cells may play a critical role in the microenvironment of mucosa-draining LNs. Our analysis of the marker profile of nodular B cells suggests the possible presence of two distinct subtypes, in addition to conventional follicular B cells, that may underlie the functional significance of these nodules.

First, age-associated B cells (ABCs) represent a potential cell type. These cells are unresponsive to B cell receptor or CD40 ligation but remain reactive to innate stimuli via TLR7 and TLR9 signaling. As shown by Hao et al. (40), ABCs arise from the exhaustive proliferation of mature B cells and are independent of the typical survival signals required by their precursors. Given the continual influx of pathogens into mucosa-draining LNs, ABCs may form as a result of chronic stimulation. Their survival advantage and distinct activation profile make them a compelling candidate for nodular B cells. Supporting this, similar B cell arrangements have been observed in the salivary glands of aged mice in the context of Sjögren’s disease. Bagavant et al. (145) reported a strong correlation between age and ABC accumulation in salivary tissue. In our own three-dimensional analysis of mandibular LNs in 73-week-old mice, we observed a double increase in the number of nodules compared to young mice, suggesting a potential age- and location-related functional link which is consistent with observations in other mucosal tissues.

Second, a distinct B cell population has been identified in the peripheral blood of chronic hepatitis C patients, characterized by the marker profile IgD^+^, IgM^+^, CD80^-^, and CD27^–^ (56), which closely resembles that of the nodular B cells described in this study and may correspond to a specific subpopulation of memory B cells. Further, IgM^+^ and IgD^+^ memory cells fall into the PD-L2^−^, CD80^−^ category, while not expressing these markers above naïve levels. This naïve-like memory subset lacks the elevated costimulatory molecules seen on more activated memory B cells (17) and are thought to develop through a germinal center independent pathway (46). Further, these IgM^+^ memory cells often make up a substantial fraction of the total memory pool in mice. For instance, Reynaud et al. (57) found that long-term memory cells were maintained by two main subsets, one IgG^+^ and one IgM^+^, with the latter expressing both IgM and IgD. Unswitched IgM^+^ memory B cells occupy unique and multifaceted immune functions distinct from the classical IgG^+^ memory B cells. Especially in line with mucosal immunity, so-called innate-like IgM^+^ memory B cells arise from spontaneous and chronic germinal center reactions within mucosal tissues such as Peyer’s patches and continuously replenish systemic compartments (146, 147). Their broad cross-reactivity allows them to effectively respond to mucosal commensals and pathogenic bacteria through glycan epitopes, even if they have not encountered these species yet (46, 147, 148). Moreover, their high avidity enables them to cope more efficiently with variant or mutating pathogens like *Plasmodium* species or diverse mucosal viruses (149–151). Thus, these innate-like IgM^+^ memory B cells appear critical for maintaining mucosal immune homeostasis. In this context, the medullary B cell nodules observed in mucosa-draining lymph nodes in this study may represent a structural niche of similar innate-like memory capacity, supporting rapid, cross-reactive IgM production to help maintain oral mucosal homeostasis. However, future studies will be required to definitively assess their memory-like potential. In contrast, we argue that canonical class-switched memory B cells are unlikely to be part of the nodule microenvironment due to the lack of associated class-switched Ig’s and PD-L2.

As a final note, a possible pathological explanation like hyperplasia warrants consideration. Hyperplasia refers to an increase in a specific cell population, typically involving the expansion of an existing cell type. In LNs, plasma cell hyperplasia is well documented, particularly in mandibular LNs due to their role in draining oral mucosa (136). Unlike plasma cell hyperplasia, which involves plasma cell accumulation throughout the medulla and often alters LN architecture, the nodules in question consist of B cells and remain morphologically distinct from surrounding tissue. Additionally, the overall structure of the LN is preserved, which contrasts with the architectural disruption commonly associated with hyperplastic processes (152). Therefore, we contend that these nodules are unlikely to represent plasma cell hyperplasia or a related pathological condition.

## Conclusion

In this study, we report the discovery and detailed characterization of medullary B cell-rich nodules as previously unrecognized, structurally distinct lymphoid compartments in murine mucosa-draining lymph nodes (LNs). These nodules are defined by their spherical morphology, consistent medullary localization, and enrichment of unswitched, non-proliferative CD45R^+^ B cells. Unlike classical B cell follicles, nodules lack germinal center features, do not express activation markers, and are anatomically segregated from T cell zones. Their emergence postnatally, increase with age, and confinement to mucosa-draining LNs suggest that they are a product of sustained, low-grade antigenic stimulation in mucosal environments. Functionally, nodules appear to represent a quiescent B cell niche, possibly supporting homeostasis, tolerance, or memory-like readiness. Their consistent association with medullary high endothelial venules and antigen-handling stromal cells, such as follicular dendritic cell-like and marginal reticular cell-like populations, supports their role in antigen surveillance. Proteomic and histological data argue against plasma cell hyperplasia, instead highlighting the presence of potential B cell subsets including age-associated, or naïve-like memory B cells. Finally, the preservation of nodules and the absence of lipomatosis in aged mandibular LNs suggest that these structures may actively contribute to long-term structural integrity and immune readiness through persistent B cell-stromal interactions. These findings redefine the medullary compartment as a functionally organized microenvironment and raise new questions about the role of non-follicular B cell aggregates in mucosal immunity, immune aging, and lymphoid tissue remodeling.

## Data Availability

The mass spectrometry proteomics data have been deposited to the ProteomeXchange Consortium via the PRIDE (153) partner repository with the dataset identifier PXD066656. The corresponding proteomics data analysis is provided in the **Supplementary Material**. Frame-by-frame visualizations of the synchrotron radiation-based microcomputed tomography datasets are also included in the **Supplementary Material** as compressed video files. Further inquiries can be directed to the corresponding authors.
