# Supplementary material for: Follicle-like niches outside the cortex? 3D phase-contrast µCT revealed medullary B cell nodules in mucosa-draining lymph nodes
Source: Front Immunol. 2025 Nov 19;16:1674997. doi: 10.3389/fimmu.2025.1674997 (PMC12672535; doi:10.3389/fimmu.2025.1674997)
Supplement: Supplementary file 1 [file Image1.pdf]

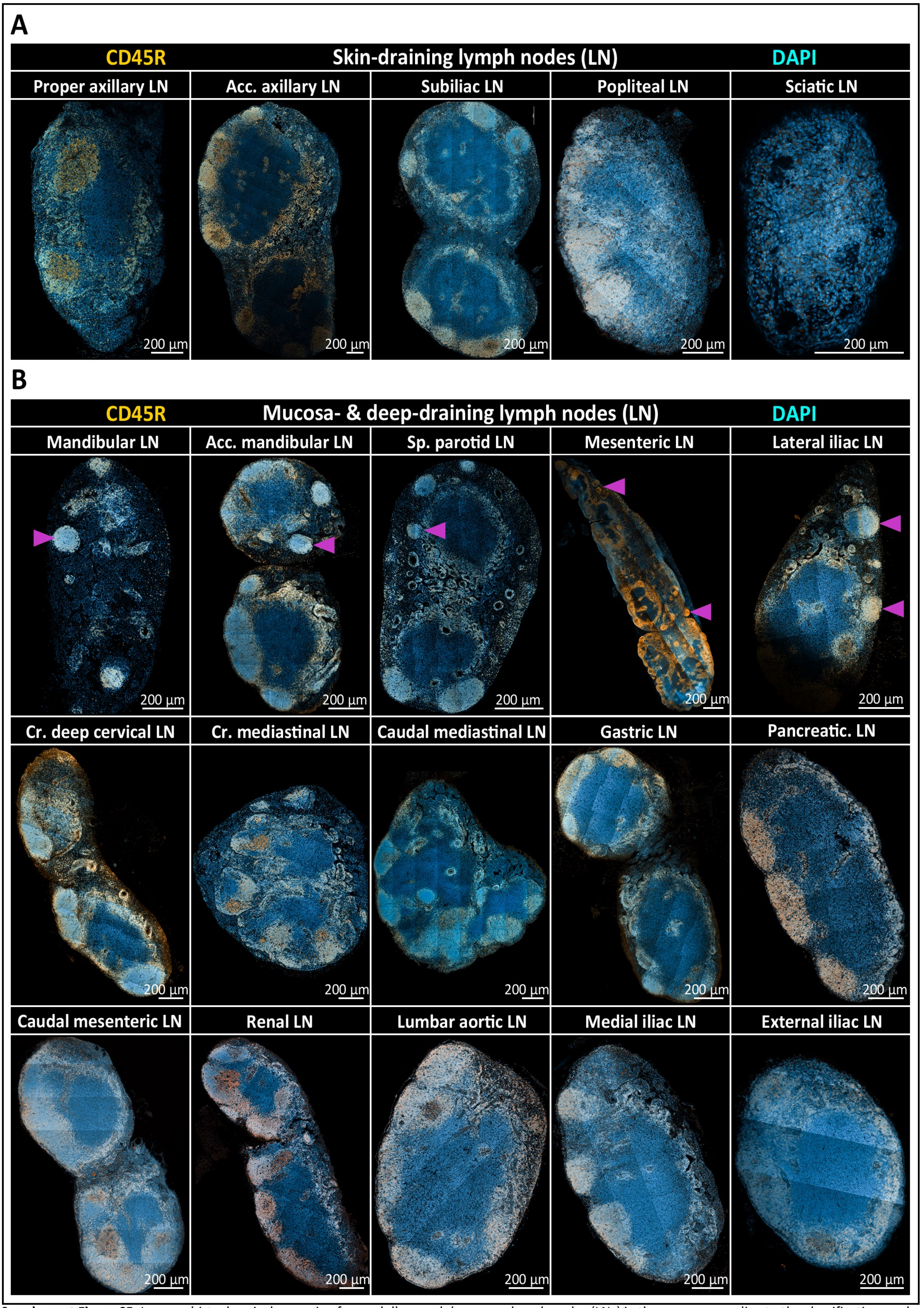

Supplement Figure S5: Immunohistochemical screening for medullary nodules across lymph nodes (LNs) in the mouse according to the classification made by Van den Broeck et al. (2006). **(A,B)** The LNs were fixed, embedded and serially sectioned at 100 µm thickness to ensure accurate identification of medullary nodules and to avoid misidentification with follicular cross sections. The tracheobronchial LN could not be reliably dissected under steady state conditions and the jejunal and colic LN are summarized under the term 'mesenteric LN' due to their clustered chain-like arrangement. Medullary nodules were visualized with anti-CD45R (orange) and nuclei were counterstained with DAPI (blue). Representative sections from four independent screenings are shown. **(A)** Medullary nodules were completely absent in skin-draining lymph nodes. **(B)** In contrast, they were detected in multiple mucosa- and deep-draining lymph nodes including the mandibular, accessory mandibular, superficial parotid, mesenteric and lateral iliac LN (magenta arrowheads). Acc.=accessory, cr.=cranial, pancreatic.=pancreaticoduodenal, sp.=superficial.
