# Supplementary material for: Follicle-like niches outside the cortex? 3D phase-contrast µCT revealed medullary B cell nodules in mucosa-draining lymph nodes
Source: Front Immunol. 2025 Nov 19;16:1674997. doi: 10.3389/fimmu.2025.1674997 (PMC12672535; doi:10.3389/fimmu.2025.1674997)
Supplement: Supplementary file 2 [file Image2.pdf]

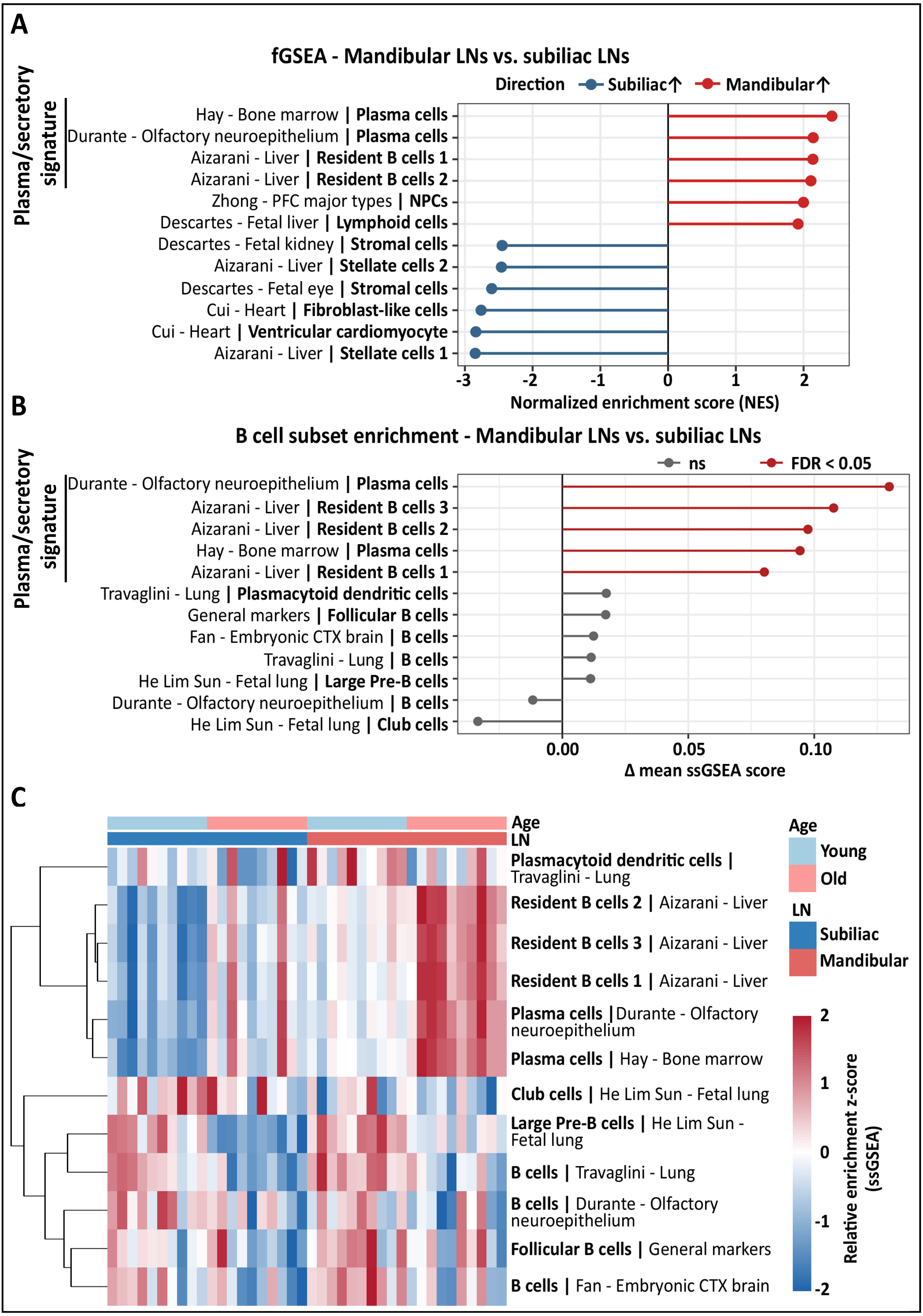

Supplement Figure S6: Marker-based enrichment of B-cell programs in mandibular vs. subiliac lymph nodes (LNs). **(A)** Pre-ranked gene set enrichment analysis (fGSEA) on limma-derived t-statistics identified significant enrichment of plasma/secretory cell programs (NES > 0, FDR < 0.05), whereas subiliac LNs displayed relative enrichment for stromal and non-immune signatures. Notably, the gene sets labelled as resident B cells in MSigDB overlapped strongly with secretory genes, and therefore reflect a plasma/secretory program rather than clear resident memory B cells. **(B)** Single-sample GSEA (ssGSEA) using MSigDB C8 gene sets and curated B cell panels confirmed the preferential enrichment of plasma cell and secretory signatures in mandibular LNs (Wilcoxon test with FDR correction < 0.05). However, the analysis could not resolve a distinct nodule-specific program. **(C)** Heatmap of ssGSEA z-scores for enriched B cell related gene sets, stratified by site and age. Enrichment of plasma/secretory cell programs was more pronounced in mandibular LNs and further increased with age.
