# Supplementary material for: Follicle-like niches outside the cortex? 3D phase-contrast µCT revealed medullary B cell nodules in mucosa-draining lymph nodes
Source: Front Immunol. 2025 Nov 19;16:1674997. doi: 10.3389/fimmu.2025.1674997 (PMC12672535; doi:10.3389/fimmu.2025.1674997)
Supplement: Supplementary file 3 [file Table1.pdf]

Supplement Table S1: Summary of the mouse and lymph node details for each experiment conducted in this study.

Experiment	Mouse count	Sex	Age	Lymph nodes
SRμCT	1	Female	17 weeks	2 mandibular, 1 mesenteric, 1 popliteal
	1	Female	73 weeks	2 mandibular, 1 mesenteric, 2 popliteal
Nodule screening	4	Female	15-20 weeks	8 mandibular
Timeline	4	Female	7 days	8 mandibular
	4	Female	14 days	8 mandibular
	4	Female	28 days	8 mandibular
	2	Female	56 days	4 mandibular
Proteomics	5	Female	17 weeks	10 mandibular, 10 subiliac
	5	Female	45-50 weeks	10 mandibular, 10 subiliac
